# White Matter Hyperintensities and Clinical Phenotype in Late-Onset Psychiatric Disorders: A Multidimensional Clinical-Neuroimaging Study

**DOI:** 10.3390/neurolint18060105

**Published:** 2026-05-26

**Authors:** Tânia Silva, Cesar Nunes, Andreia Ribeiro, Isabel Santana, Joaquim Cerejeira

**Affiliations:** 1 Department of Psychiatry, Unidade Local de Saúde de Coimbra, University Hospital, 3000-075 Coimbra, Portugal; 2 University of Coimbra, Faculty of Medicine, 3000-370 Coimbra, Portugal; 3 University of Coimbra, Coimbra Institute for Clinical and Biomedical Research (iCBR), Faculty of Medicine, 3000-370 Coimbra, Portugal; 4 University of Coimbra, Center for Innovative Biomedicine and Biotechnology (CIBB), 3004-504 Coimbra, Portugal; 5 Department of Neuroradiology, Unidade Local de Saúde de Coimbra, University Hospital, 3000-075 Coimbra, Portugal; 6 Department of Neurology, Unidade Local de Saúde de Coimbra, University Hospital, 3000-075 Coimbra, Portugal

**Keywords:** late-onset psychiatric disorders, white matter hyperintensities, cerebral small-vessel disease, neuropsychiatric, vascular cognitive impairment

## Abstract

Background: White matter hyperintensities (WMHs) have been implicated in late-onset psychiatric disorders, but their contribution to this clinical phenotype remains insufficiently understood. Methods: We conducted a cross-sectional transdiagnostic study of 90 consecutively admitted acute patients with schizophrenia, bipolar disorder (BD), and major depressive disorder (MDD) meeting the predefined inclusion criteria. Patients with a late onset were compared to earlier onset (EO) psychiatric patients. Late onset was defined as the median age of the disorder onset of the sample (≥40 years). Multidimensional clinical, cognitive, psychomotor, metabolic, and neuroimaging data were evaluated (WHM burden and cerebral atrophy), and a cognitive-psychopathologic composite index was derived. Correlations and sensitivity analysis were performed. A multivariable linear regression was performed to assess the independent effects of age, vascular risk factors, and WMH severity on cognitive performance and psychiatric symptoms. Results: In patients with LO psychiatric disorders, greater WMH burden was significantly associated with poorer global cognition and specific cognitive domains, lower delusional symptoms severity, and greater suicidal thoughts/behavior intensity. These associations were markedly weaker or not present in EO patients. The regression model explained 36.5% of the variance in the cognitive-psychopathologic composite index. After adjusting for age and cumulative risk factors, Fazekas was the only significant independent predictor (β = −0.495, *p* = 0.001). Conclusions: WMH burden was associated with differences in clinical characteristics in LO psychiatric disorders, including cognitive and neuropsychiatric symptoms. Our findings support a possible vascular-neuropsychiatric interaction in LO phenotypes.

## 1. Introduction

A significant group of psychiatric patients is considered to have late onset (LO) disorders, having distinct clinical characteristics and associations with biological factors [1,2].

Psychotic episodes with LO are more frequent in women, and these patients present with more severe paranoid symptoms and persecutory delusions [2,3]. Patients with LO depression more frequently present hypochondriacal ideation, early insomnia, agitation, preoccupation with guilt, and delusional ideation [4,5].

LO disorders, both psychotic spectrum and affective disorders, have lower genetic risk [2,3,4,5] and higher rates of cognitive dysfunction and subsequent dementia [2,4,5,6].

White matter hyperintensities (WMHs) of presumed vascular origin, resulting from cerebral small-vessel disease, are more prevalent and have a higher burden in depression, bipolar disorder, and psychotic disorders with LO [2,4,7,8,9,10].

The distinctive clinical and neurobiological features of LO disorders support the hypothesis that these are phenotypic variations, which may involve different pathophysiological mechanisms. Phenotypic characterization and the identification of biomarkers, such as WMH, in LO psychiatric disorders can contribute to a more precise treatment strategy, a better understanding of the disease trajectory, and a more precise outcome prediction.

Scientific research on LO psychiatric disorders is fragmented, focuses on a single diagnosis, uses a single gradation scale, and rarely integrates multiple clinical dimensions (cognitive, psychopathology, and neurobiological factors).

The aim of this study was to investigate whether the burden and/or topography of WMHs are associated with differences in the symptom profiles of LO psychiatric disorders. To this end, we conducted a transdiagnostic investigation in a psychiatric population with major psychiatric diseases.

## 2. Methods

### 2.1. Participants

The investigators performed a cross-sectional study of patients consecutively admitted to the acute psychiatric ward of a University Hospital (Coimbra Local Health Unit) from October 2022 to October 2024, aged 45 to 75 years, who met the criteria for schizophrenia, bipolar disorder (BD), and major depressive disorder (MDD) according to the Diagnostic and Statistical Manual of Mental Disorders—5th edition (DSM-5). The exclusion criteria are presented in Supplementary Material S1.1.

The ethics committee of the University Hospital (Coimbra Local Health Unit) approved all the procedures used in this study, including the method of obtaining consent, in accordance with the Declaration of Helsinki. Study protocols and rationale were explained to the participants, and all provided written informed consent.

### 2.2. Clinical Assessment

All patients were evaluated with the Structured Clinical Interview for DSM-5—Clinician Version (SCID-5-CV) for psychiatric diagnosis by two experienced psychiatrists. Sociodemographic data, medical and psychiatric history, and family history of psychiatric disorders were collected through a questionnaire. Psychopathological symptoms were operationalized with the Neuropsychiatric Inventory (NPI), 12-item version, which assesses delusions, hallucinations, agitation/aggression, depression, anxiety, elation, apathy, disinhibition, irritability, aberrant motor behavior, and sleeping and eating disorders [11,12,13]. Suicide risk was evaluated with the Suicide Behaviors Questionnaire-Revised (SBQ-R) [14,15], and functioning was evaluated with the Global Assessment of Functioning (GAF) scale [16].

Cognitive assessment occurred after achieving clinical stability and included the Mini Mental Status Examination (MMSE) [17] and Montreal Cognitive Assessment (MoCA) [18]. The psychomotor assessment included the Finger tapping test (FFT) [19,20,21,22] and the Short Parkinson evaluation scale (SPES) [23,24].

Additional vascular-metabolic markers included lipid profile (Total Cholesterol; HDL-C; LDL-C; TG), HbA1c, weight, height, and blood pressure.

### 2.3. Neuroimaging Assessment

The neuroradiologic assessment was performed by a team of two neuroradiologists who independently classified the images, blind to the diagnosis. Differences in score attribution were resolved by discussion. The neuroimaging protocol is described in Supplementary Material S1.2.

WMH burden assessment was performed with the Fazekas scale and the Modified Scheltens rating scale (MSRS). The Fazekas scale attributes a grade to periventricular white matter and deep white matter depending on the size and confluence of lesions [25].

The MSRS classifies subregions separately (periventricular WMH: occipital caps, frontal caps, and lateral ventricles bands; deep WMH: frontal, parietal, occipital, and temporal lesions) [26].

### 2.4. Statistical Analysis

Statistical analysis was performed using IBM SPSS Statistics for Macintosh, version 29.0 (IBM Corp., Armonk, NY, USA). Descriptive analysis was used to determine the clinical characteristics of the participants.

The median age of psychiatric disorder onset of the sample (40 years) was used as the cutoff value for an LO disorder in this sample.

Patients with an LO disorder were compared with patients with a disorder onset before 40 years. Chi-square and Mann–Whitney tests were used to compare qualitative and quantitative variables, respectively. Correlations between WMH characteristics and other variables were investigated using Kendall’s tau correlation analysis. The Benjamini–Hochberg FDR (False Discovery Rate) procedure was applied within families of tests defined by categories (cognition, neuropsychiatric, motor, neuroimaging, and metabolic/vascular) to control for type I error resulting from multiple comparisons. The significance level adopted was *p* < 0.05 after correction. A sensitivity analysis was performed using alternative age-onset thresholds (≥50 years and ≥60 years) to assess the robustness of the 40-year age-of-onset threshold (Supplementary Material S10).

We also performed a sensitivity analysis to analyze potential diagnostic-specific effects in the LO group. Since the statistical power was very reduced due to small subgroup sizes, the analysis was exploratory and intended to assess the qualitative consistency with emphasis on directionality (Supplementary Material S9) [27,28].

To control for potential confounders known to be highly correlated with WMHs, including age and vascular risk factors, a multiple linear regression model was conducted in the subgroup of patients with a disease onset <40 years and in the subgroup of patients with a disease onset ≥40 years to examine whether age, vascular burden, and WMH burden was associated with the cognitive-psychopathologic profile of the patients with a disease onset ≥ 40 years.

We created a simple standardized “cognitive-psychopathologic composite index” to reflect the late onset phenotype according to the correlation analysis results. This composite was created by standardizing and averaging cognitive and psychopathological measures related to the late onset phenotype—MoCA visuospatial/executive, MoCA orientation, MoCA memory, NPI delusions, SBQ-R, and FTT score. To account for the cumulative effect of vascular risk factors, a vascular composite score described elsewhere was used [29]. The variables included in the score were diabetes, hypertension, hypercholesterolemia, smoking, alcohol abuse, body mass index, and blood pressure. All variables were inspected for linearity, normality of residuals, and multicollinearity. The statistical significance was set at *p* < 0.05.

## 3. Results

### 3.1. Sample Characteristics

A total of 128 patients were recruited, but only 90 patients were included in the final sample. Thirty-five subjects were excluded due to episodes of delirium during hospitalization, diagnosis of Parkinson’s disease, signs of stroke on MRI, and signs of trauma on MRI. Eight patients declined to participate in the study, corresponding to a nonresponse rate of 8.16%.

The mean age of the sample was 59.83 (SD 7.72) years, composed mainly of women (71.1%), with 100% Caucasians. Most patients had dyslipidemia (82.2%), more than half of the patients had HT (56.7%), and 21.1% had DM. Smoking was identified in 21.1% of patients. The mean body mass index (BMI) was above normal at 27.01 kg/m^3^ (SD = 4.45). Almost all patients (90%) were being treated with atypical antipsychotics, while only 10% were being treated with typical antipsychotics. Furthermore, 90% of patients were using benzodiazepines. Regarding mood stabilizers, 12.2% were being treated with lithium and 25.6% with valproate/valproic acid. Patients undergoing antidepressant treatment were mostly treated with SSRI, and psychostimulants were prescribed to 4.4% of patients (Supplementary Material S2).

Patients with a LO and EO had similar mean ages and did not differ in the incidence of vascular risk factors. Patients with an LO psychiatric disorder showed lower scores on the MoCA memory subtest, had fewer psychiatric hospitalizations, and were less sedentary than patients with an EO (Table 1).

### 3.2. Cognitive Correlation with WMH

In patients with an LO, a higher Fazekas grade was associated with worse performance on MoCA. In the analysis of the MoCA subtests, moderate negative correlations were found between the Fazekas grade and the visual/executive, abstraction, memory, and orientation domains. The orientation domain of MoCA was a consistent association, given that its score showed a moderate negative correlation with both the Fazekas scale and the MSRS total and subscales (Table 2; Supplementary Materials S5–S7). These correlations were found only in patients with LO, indicating that greater WMH lesion burden was associated with poorer orientation performance in patients with LO. We found no correlation between WMH lesion burden and FAB score in LO patients (Table 2).

### 3.3. Psychomotor Correlates

In patients with LO, greater WMH burden was associated with slower motor velocity and extrapyramidal signs. The FTT showed a moderate negative correlation with the Fazekas grade (τ = −0.319, *p* = 0.030), and the SPES score showed a moderate positive correlation with the Fazekas grade and the MSRS score in patients with LO (Table 2).

### 3.4. Neuropsychiatric Symptoms

Regarding psychiatric history, a moderate positive correlation was found between the number of psychiatric hospitalizations and the MSRSd temporal lesions score in patients with a LO (Supplementary Material S5).

Deep WMH lesions were associated with a lower score on the NPI, in particular, a lower score in the delusions domain and the irritability domain (Supplementary Material S5). Upon analyzing the different deep sub-regions, we found that deep parietal lesions were associated with a lower score in NPI, the delusions domain, the agitation domain, and the irritability domain. Similarly, deep frontal lesions were associated with a lower score in the delusions domain and the irritability domain (Supplementary Material S6).

The delusions domain’s negative correlation was the most consistent finding, as its score showed a moderate negative correlation with the burden of WMH lesions, measured by both rating scales (Table 2).

Regarding suicidal thoughts/behaviors in patients with LO, the SBQ-R score was associated with an increased deep lesion burden, particularly with deep parietal lesions and deep temporal lesions.

### 3.5. Neuroimaging—Cortical Atrophy

Frontal atrophy (Supplementary Materials S5–S7) was associated with the burden of WMH lesions in both LO and EO, but the correlation was stronger in LO patients.

### 3.6. Multiple Linear Regression

In patients with an onset < 40 years, the model did not significantly predicted the cognitive-psychopathologic composite index (F(3, 40) = 1.393, R^2^_a_ = 0.027, *p* = 0.259; Age: B = 0.005, 95% CI [−0.012, 0.022], β = 0.104, *p* = 0.536; Vascular burden: B = −0.081, 95% CI [−0.175, 0.012], β = −0.302, *p*= 0.086; Fazekas grade: B = −0.038, 95% CI [−0.248, 0.170], β = −0.064, *p* = 0.710).

In patients with an onset ≥ 40 years, the linear regression model was significant F(3, 42) = 9.625, R^2^_a_ = 0.365, *p* < 0.001) and explained 36.5% of the variance of the cognitive-psychopathologic composite index. The analysis of the regression coefficients revealed that the Fazekas grade was independently associated with lower cognitive-psychopathologic composite score (B = −0.364, 95% CI [−0.580, −0.149], β = −0.495, *p* = 0.001), while age (B = −0.020, 95% CI [−0.043, 0.003], β = −0.245, *p* = 0.088) and vascular burden (B= 0.026, 95% CI [−0.071, 0.122], β = 0.068, *p* = 0.592) showed a non-significant association (Supplementary Material S11). The standardized effect size indicated that WMH burden accounted for a proportion of the variance in the cognitive-psychopathological performance. After adjustment for age and vascular risk factor burden, a one-standard-deviation increase in WMH severity was associated with an approximately half-standard-deviation decrease in the cognitive-psychopathologic composite index.

### 3.7. False Discovery Rate Control

After adjusting for multiple testing, the association between WMH burden and cognitive deficits, motor symptoms, delusions, metabolic markers (glycate hemoglobin), and frontal atrophy (GCA-F) persisted. These findings support a possible role of cerebral small-vessel disease in the clinical expression of LO psychiatric disorders (Supplementary Material S8).

### 3.8. Diagnostic-Specific Sensitivity Analysis

Although statistical significance varied among diagnostic subgroups, the overall direction of associations between WMHs and the other variables was consistent across all diagnoses (Supplementary Material S9).

### 3.9. Age Onset Sensitivity Analysis

The sensitivity analysis revealed comparable patterns of association between WMHs and clinical variables using alternative age-of-onset thresholds (≥50 and ≥60 years). Using a threshold of ≥50, the pattern of associations did not differ between WMH burden and other variables. All major associations observed with a threshold of ≥40 persisted. Using a threshold of ≥60, the associations were preserved in terms of direction, but the statistical significance of the correlations was significantly reduced due to sample size (Supplementary Material S10).

## 4. Discussion

**Figure 1:**
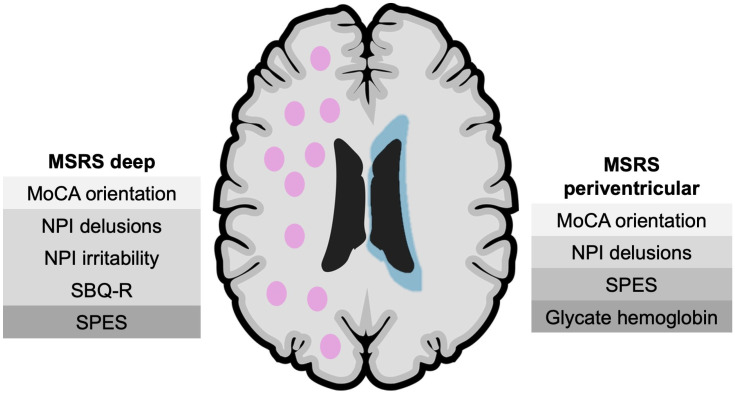
Significant correlations found between the Fazekas grade, Modified Scheltens rating scale (MSRS) score, and multiple clinical variables in patients with LO. MoCA—Montreal cognitive assessment, NPI—neuropsychiatric inventory, SPES—short Parkinson’s evaluation scale. Pink—deep lesions, blue—periventricular lesions.

**Figure 2:**
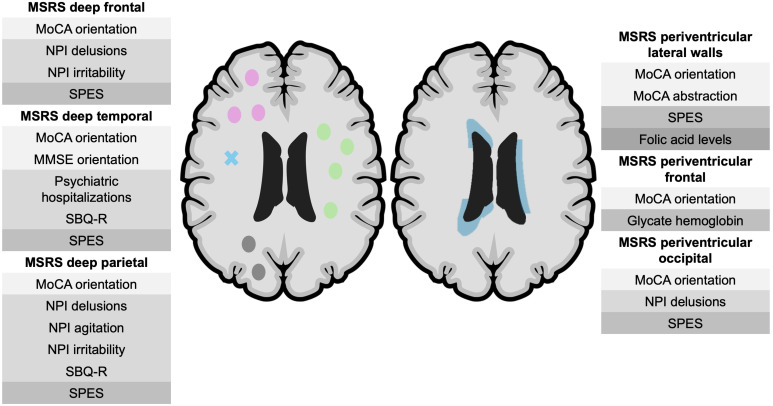
Significant correlations found between sub-regions (MSRS subscores) and multiple clinical variables in the patients with LO. MoCA—Montreal cognitive assessment, MSRS—Modified Scheltens ratting scale, NPI—neuropsychiatric inventory, SBQ-R—suicide behaviors questionnaire revised, SPES—short Parkinson’s evaluation scale. On the left: pink—frontal deep lesions, blue cross—deep temporal lesions, green—deep parietal lesions. On the right: blue—periventricular lesions.

This study addresses neurobiological aspects of LO psychiatric disorders, investigating the association between cerebral small-vessel disease and clinical factors. Figure 1 and Figure 2 present diagrams of the most relevant findings in the study. Despite their distinctive clinical features, LO psychiatric disorders remain understudied, and clinical guidelines do not differentiate the LO forms. Our study contributes to the characterization of LO psychiatric disorders as presenting distinct clinical and neurobiological features, rather than a simple delay in disease onset.

Although patients with major psychiatric disorders are reported to have a higher burden of WMH, the role of these lesions, especially in LO disorders, is poorly understood [2,4,7,8,9,10].

Our study demonstrated a correlation between WMHs and specific cognitive domains and psychopathological symptoms. In the multivariate analysis, after accounting for age and vascular risk factors, WMH severity remained a significant factor associated with a profile defined by low cognitive performance, low severity of psychotic behaviors, and higher intensity of suicide behaviors/thoughts (cognitive-psychopathologic composite) in the LO subgroup.

### 4.1. WMHs and Cognitive Impairment

In patients with an LO psychiatric disorder, higher lesion load was correlated with worse performance on the MoCA (total and visuospatial/executive, memory, abstraction, and orientation subdomains). This was a finding consistent with the scientific literature, which reports the strong association between WMHs and cognitive impairment and dementia [30]. Cerebral small-vessel disease is known to involve demyelization and axonal loss, processes that have been associated with disruption of long-range white matter circuits supporting higher-order cognition, including abstraction and orientation [30]. LO psychiatric disorders, in particular LO schizophrenia, are commonly associated with executive dysfunction, and along with memory, these domains appear to be more affected in late presentations [31,32].

### 4.2. WMHs and Neuropsychiatric Symptoms

Patients with LO psychiatric disorder showed an inverse correlation between the severity of delusions and the burden of WMH. Delusions in primary psychotic disorders are characterized by high levels of internal coherence, thematic complexity, and temporal stability. These features rely on a preserved cognitive performance, particularly executive control, memory, and abstract reasoning. WMHs are known to be strongly associated with cognitive impairment, particularly involving executive dysfunction, slow processing speed, attention deficits, and memory impairment [30,33]. Cognitive impairment affects thought organization and reasoning, which can impair the construction, complexity, and persistence of delusional thoughts. This can lead to less structured, simpler, less persistent, and confusion-driven psychotic symptoms, in contrast to psychotic disorders not associated with WMH, which are more complex, bizarre, and systematized [34].

Research in the field of psychotic disorders has consistently revealed the existence of subgroups of patients characterized by a specific aggregation of psychiatric and cognitive symptoms. The most notable example is the two-syndrome classification of schizophrenia proposed by Timothy J. Crow in the 1980s. More recent studies report subtypes of psychoses that incorporate structural brain characteristics, even allowing for an identification based on MRI. The importance of identifying subgroups lies in the different responses to treatment and evolution, as well as possible different pathophysiologic mechanisms [35].

An alternative explanation could be the underestimation of the severity of psychotic symptoms since patients with LO delirium appear to have greater cognitive impairment. Reduced awareness of the disorder and a decreased ability to recall delusional experiences may potentially lead patients to underreport symptoms.

Greater deep WMH lesion burden was associated with more intense suicidal thoughts and behavior in LO patients, which is consistent with the scientific literature reporting a higher WMH burden in suicide attempters [36]. Also, greater cognitive impairment has been reported in late-life depressive patients with suicide attempts when compared with non-attempters [37].

Our findings were consistent with the literature, supporting a higher suicide risk in patients with WMH. The link between WMHs and suicide can be explained by a neural network disconnection caused by the disruption of association fibers of ventral/dorsal prefrontal circuits and their connections to limbic and posterior regions underlying emotional dysregulation and reduced stress control [38].

### 4.3. WMHs and Motor Symptoms

Psychomotor performance (FTT) and extrapyramidal symptoms (SPES) showed an association with WMH load in patients with LO disease. The value of this result is limited since the confounding effect of the medication cannot be assessed due to the widespread use of psychopharmacological medication.

### 4.4. WMHs and Frontal Atrophy

Patients with an LO psychiatric disorder showed an association between frontal atrophy and WMH burden, supporting a possible convergence of vascular and neurodegenerative processes. Growing evidence indicates that the pathogenic process of cerebral small-vessel disease is not confined to white matter. Cerebral small-vessel disease is associated with cortical degeneration, as WMHs have been consistently linked to cortical thinning, particularly in the frontal and parietal regions [39]. Cerebral small-vessel disease may accelerate regional cortical vulnerability to degeneration by interacting with neurodegenerative processes rather than acting as an isolated pathogenic process. Corroborating this hypothesis, the patterns of cortical thinning associated with WMHs overlap with the atrophy patterns observed in normal aging and Alzheimer’s disease [39].

### 4.5. WMH Burden and Glycemic Dysregulation

Glycate hemoglobin showed a correlation with WMHs, particularly in periventricular regions. This finding was consistent with the strong association of WMHs with diabetes status and impaired glucose metabolism [40,41].

### 4.6. Early Onset vs. Late Onset: Distinct Biological and Clinical Profiles

Patients with an EO showed fewer associations between WMHs and clinical factors. In addition to the distinct clinical characteristics, our study supports a possible vascular-neuropsychiatric interaction in LO phenotypes.

### 4.7. Methodologic Aspects, Limitations, and Future Directions

The sample had an adequate size to allow a subgroup analysis of patients with LO psychiatric disorders with sufficient statistical power. However, the studied population consisted exclusively of acutely ill patients with major psychiatric disorders, which limits the generalizability of the findings to broader psychiatric populations. In addition, some relevant variables, including physical activity and family history of psychiatric disorders, were based on self-report information and therefore are subject to reporting bias.

A limitation of this study was the transdiagnostic approach. The purpose of the transdiagnostic approach was to replicate real-life clinical practice and explore neurobiological factors across disorders. However, a transdiagnostic approach may be a limitation due to potential diagnostic-specific effects. Despite this concern, the sensitivity analysis stratified by diagnosis revealed consistency, giving robustness to the results. Formal interaction analysis testing diagnosis × WMH or onset group × WMH interactions was not performed, given the modest sample size and further reduction in statistical power with the diagnostic stratification. Larger samples and a longitudinal design will be required to formally assess interaction effects and diagnostic specificity.

Also, a potential limitation was the definition of LO psychiatric diseases. Since there is no universally accepted age limit for the onset of psychiatric disorders, our selection was based on the median of the sample. The sensitivity analysis using alternative age-at-onset thresholds (disease onset at ≥50 and ≥60 years) revealed comparable patterns of association between WMHs and clinical variables, showing the consistency of results.

Regarding neuroimaging data acquisition, visual semi-quantitative rating scales (Fazekas scale and MSRS) were used. These instruments are widely validated and have the advantage of being readily applicable in routine clinical practice. Lesion classification is categoric, being restricted to a range of values, which reduces sensitivity for detecting subtle associations [42].

Moreover, the lesions progress along a continuum, whereas the severity gradation across scale categories is neither linear nor proportional, resulting in imprecise burden classification. Lastly, reproducibility issues related to interobserver disagreement must be considered [42,43].

Regarding the cognitive assessment, the MMSE showed a poor correlation with WMH burden. This finding is consistent with the known limited sensitivity of the MMSE for detecting mild cognitive impairment and cognitive dysfunction not predominantly related to Alzheimer’s disease. The short-memory and visuospatial tasks included in the MMSE have low complexity, rendering the instrument less capable of identifying subtle cognitive deficits, particularly those involving executive functions [44].

Given that WMHs are primarily associated with subcortical damage and executive dysfunction [30], the MMSE appears to be an inappropriate screening tool for populations with microvascular-related cognitive impairment. In contrast, the MoCA demonstrates greater sensitivity and superior discriminatory power, making it a more appropriate tool for cognitive screening of preclinical or mild cognitive impairment patients [44].

Medication exposure represents a potential confounder in both WMHs and motor findings. However, because our sample includes only acutely ill patients, all participants were undergoing psychopharmacological treatment, with 90% being treated with second-generation antipsychotics. The independence test revealed no statistically significant association between the subgroups and pharmacological treatment, except for the use of lithium, which was more frequent in patients with a disease onset <40 years. Due to the high prevalence and homogeneity of medication exposure, formal comparisons were not feasible.

Finally, future longitudinal studies will be essential to determine how WMHs predict clinical worsening, cognitive decline, or functional deterioration in patients with LO psychiatric disorders.

## 5. Conclusions

In conclusion, this study supported a potential association between WMH burden and cognitive and psychopathological features in LO psychiatric disorders across selected major psychiatric disorders. These associations persisted after adjusting for age and cumulative vascular risk factors, reinforcing the view that WMHs were not incidental findings in this population, but rather may be correlated with variations in psychiatric phenotype. Given the correlational cross-sectional design, all findings should be interpreted as associative and do not support causal or mechanistic inferences about the role of WMHs in psychiatric phenotypes. Instead, they highlight the need for further research, including longitudinal studies.

## Figures and Tables

**Table 1:** Comparison between individuals with disease onset before age 40 and individuals with disease onset at age 40 or older. Stat.—statistic test, ^(1)^ Student’s *t*-test, ^(2)^ Chi-squared test, ^(3)^ Mann-Whitney U test, M—mean, SD—standard deviation, MDD/BD—major depressive disorder and bipolar disorder.

Variables	Onset < 40 y N = 44	Onset ≥ 40 y N = 46	Stat.	Sig.
Age, M (SD)	59.09 (8.42)	60.54 (6.99)	T = 0.89 ^(1)^	0.37
Education level, %			χ^2^ = 3.62 ^(2)^	0.082
≤12 years	52.3	71.7		
>12 years	47.7	28.3		
Marital status, %			χ^2^ = 0.75 ^(2)^	0.68
Single	15.9	19.6		
Married/partnership	59.1	50		
Widowed/divorced	25	30.4		
Medical history, %				
HT	50	63	χ^2^ = 1.55 ^(2)^	0.15
DM	18.2	23.9	χ^2^ = 0.44 ^(2)^	0.34
Dyslipidemia	81.8	82.6	χ^2^ = 0.010 ^(2)^	0.57
Tobacco abuse	22.7	19.6	χ^2^ = 0.13 ^(2)^	0.45
Physical activity—Sedentary, %	65.9	37	χ^2^ = 7.54 ^(2)^	0.007
Body mass index, M (SD)	27.92 (4.25)	26.13 (4.50)	T = −1.94 ^(1)^	0.055
Obese, %	31.8	19.6	χ^2^ = 1.77 ^(2)^	0.23
Sleep apnea risk, %			χ^2^ = 1.61 ^(2)^	0.44
Low risk	59.1	54.3		
Increased risk	34.1	30.4		
Sleep apnea diagnosis	6.8	15.2		
Age of psychiatric disorder onset, M (SD)	27.72 (6.71)	52.06 (9.52)	T = 14.06 ^(1)^	<0.001
Psychiatric hospitalizations, M (SD)	3.52 (4.03)	2.13 (1.47)	U = 760 ^(3)^	0.033
Family history of psychiatric disease, %				
Dementia before 65 years	9.1	6.5	χ^2^ = 0.20 ^(2)^	0.47
Dementia at 65 or more	20.5	28.3	χ^2^ = 0.74 ^(2)^	0.26
MDD/BP	45.5	32.6	χ^2^ = 1.56 ^(2)^	0.15
Schizophrenia/psychosis spectrum	18.2	8.7	χ^2^ = 1.75 ^(2)^	0.15
Suicide	13.6	10.9	χ^2^ = 0.16 ^(2)^	0.46

**Table 2:** Kendall’s tau correlation analysis between multiple variables of the subgroup of patients with LO. τ-Kendall’s tau correlation coefficient, MSRS—Modified Scheltens rating scale, MoCA—Montreal Cognitive assessment total score adapted for educational level, MMSE—Mini mental state examination total score, FTT—Finger tapping test, FAB—Frontal assessment battery, SPES—Short Parkinson evaluation scale, NPI—Neuropsychiatric inventory total score, SBQ-R—Suicide Behaviors Questionnaire-Revised, MTA—Medial temporal atrophy scale, GCA-F—Global cortical atrophy/Pasquier scale frontal subscale.

	Onset ≥ 40 Years, N = 46			
	Fazekas Scale		MSRS	
Variables	Stat. (τ)	Sig.	Stat. (τ)	Sig.
MoCA	−0.437	0.002	−0.259	0.081
Visuospatial/executive	−0.311	0.035	−0.166	0.267
Naming	−0.211	0.157	−0.085	0.572
Attention	−0.030	0.838	0.120	0.426
Language	−0.197	0.187	−0.094	0.532
Abstraction	−0.358	0.014	−0.257	0.084
Memory	−0.369	0.011	−0.258	0.082
Orientation	−0.359	0.014	−0.378	0.009
MMSE	−0.275	0.063	−0.097	0.518
Orientation	−0.313	0.033	−0.162	0.280
Registration				
Attention	−0.06	0.645	0.093	0.535
Recall	−0.010	0.942	−0.040	0.787
Language	−0.011	0.942	0.080	0.593
Visual construction	−0.20	0.182	−0.183	0.223
FAB	−0.159	0.353	−0.017	0.920
FTT	−0.319	0.030	−0.124	0.411
SPES	0.433	0.003	0.402	0.006
Psychiatric hospitalizations	−0.087	0.561	0.029	0.849
NPI total	−0.242	0.104	−0.286	0.053
Delusions	−0.441	0.002	−0.383	0.008
Hallucinations	−0.209	0.161	−0.190	0.204
Agitation	−0.095	0.526	−0.166	0.267
Depression/dysphoria	0.077	0.608	−0.008	0.956
Anxiety	−0.010	0.943	−0.071	0.637
Euphoria	−0.056	0.707	−0.024	0.871
Apathy	0.160	0.285	0.050	0.738
Disinhibition	−0.141	0.349	−0.077	0.608
Irritability	−0.217	0.146	−0.286	0.053
Motor behavior	0.066	0.661	0.149	0.320
Nocturnal behavior	−0.112	0.456	−0.085	0.571
Appetite	−0.092	0.541	−0.072	0.631
SBQ-R	0.238	0.110	0.274	0.064
MTA	0.182	0.253	0.139	0.383
Koedam score	−0.089	0.554	−0.053	0.724
GCA-F	0.390	0.007	0.248	0.095
Glycate hemoglobin	0.289	0.063	0.274	0.078

## Data Availability

The data presented in this study are available on request from the corresponding author due to privacy reasons.

## Supplementary Materials

The following supporting information can be downloaded at: https://www.mdpi.com/article/10.3390/neurolint18060105/s1, Supplementary Material: S1.1—exclusion criteria, S1.2—neuroimaging protocol, Supplementary Material S2—Demographic and clinical characteristics of the sample, Supplementary Material S3—Comparison between individuals with disease onset before age 40 and individuals with disease onset at age 40 or older, Supplementary Material S4—Comparison between individuals with disease onset before age 40 and individuals with disease onset at age 40 or older, Supplementary Material S5—Kendall’s tau correlation analysis between multiple variables of the subgroup of patients with late onset, Supplementary Material S6—Kendall’s tau correlation analysis between multiple variables of the subgroup of patients with late onset, Supplementary Material S7—Kendall’s tau correlation analysis between multiple variables of the subgroup of patients with late onset, Supplementary Material S8—Benjamini–Hochberg FDR (False Discovery Rate) correction applied within families of tests defined by categories—anthropometric category, Supplementary Material S9—Sensitivity analysis. Direction and consistency of Kendall’s tau correlation analysis between multiple variables of the subgroup of patients with disease onset at age 40 or more, stratified by diagnosis (Schizophrenia spectrum disorders, Bipolar disorder, Major Depressive disorder), Supplementary Material S10—Sensitivity analysis. Kendall’s tau correlation analysis in patients with disease onset at age 50 or more and age 60 or more, Supplementary Material S11—Multiple linear regression model conducted in the subgroup of patients with a disease onset ≥ 40 years to examine whether age, vascular burden and WMH burden was associated with the cognitive-psychopathologic composite index (MoCA visuospatial/executive, MoCA orientation, MoCA memory, NPI delusions, and SBQ-R), Supplementary Material S12—Comparison of the proportion of psychopharmacological treatments between the patients with an onset before 40 years and ate 40 or more.
