# Supplementary material for: White Matter Hyperintensities and Clinical Phenotype in Late-Onset Psychiatric Disorders: A Multidimensional Clinical-Neuroimaging Study
Source: Neurol Int. 2026 May 26;18(6):105. doi: 10.3390/neurolint18060105 (PMC13304622; doi:10.3390/neurolint18060105)
Supplement: Supplementary file 1 [file neurolint-18-00105-s001.zip › neurolint-4283924-supplementary.pdf]

Supplementary material S1.1) Exclusion criteria

Patients were excluded if they:

- had a known diagnosis of monogenic forms of cerebral small vessel disease (CADASIL, CARASIL, familial cerebral amyloid angiopathy, HANAC, Fabry disease, homocystinuria, MELAS, HDLS, RVCL, Axenfeld-Rieger syndrome);
- met DSM-5 criteria for comorbid diagnosis of intellectual disabilities, autism spectrum disorders, feeding and eating disorders and alcohol/substance use disorders;
- presented a state of delirium according to clinical criteria;
- had been diagnosed with major neurological diseases (e.g., Parkinson Disease, Huntington disease, amyotrophic lateral sclerosis, epilepsy);
- met criteria for Dementia according to international standardized criteria (Alzheimer dementia, Frontotemporal dementia, multi-infarct vascular or Lewy body dementia);
- presented radiological evidence of non-vascular demyelinating syndromes, unilateral cerebral microvascular lesions or known history or neuroradiological signs of great vessel stroke;
- had clinically significant structural brain damage or CNS trauma in the past;
- had history of HIV or spirochetal infection of CNS (e.g., syphilis, borreliosis, or Lyme disease), acute medical illness, autoimmune disorders, history of cancer within the past 5 years, recent major surgery, untreated hypothyroidism or vitamin deficiency (cobalamin, folate and vitamin D);
- were under legal custody (imprisonment).

Supplementary material S1.2) Neuroimaging protocol.

- Neuroimaging protocol: 3-T Siemens Magneton Vida scanner (Munich, Germany) Magnetization-prepared rapid gradient- echo T1- weighted images (MP-RAGE) acquired in sagittal plane and T2/FLAIR images

acquired in the axial plane. FLAIR: repetition time 9000 ms, echo time 91 ms, inversion time 2500 ms, flip angle 150°, slice thickness 3 mm, field of view 256 × 256 mm. T2-weighted: repetition time 5500 ms, echo time 108 ms, 130° flip angle, slice thickness 3 mm, field of view 256 × 256 mm. T1-3D MP-RAGE (when available): repetition time 2000 ms; echo time 2.2 ms; inversion time 900 ms; flip angle 8°; parallel imaging - GRAPPA 2), voxel size 0.5 mm × 0.5 mm x 0.5; matrix size 256 × 256; number of slices 352.

Supplementary material S2.

				N=90
**Age**, M (SD)	**Male**, %	**Caucasian**, %	**Education level**, %	**Marital status**, %
59.83 (7.72)	28.9	100	4 years, 35.6	Single, 17.7
			9 years,13.3	Married/partnership, 54.4
			12 years, 13.3	Widowed/divorced, 27.8
			>12 years, 37.8	
**Medical history**, %	**BMI**, M (SD)	**OSA**, %	**Physical activity**, %	**Medication (somatic)**, %
HT, 56.7	27.01 (4.45)	Low risk, 56.7	Sedentary, 51.1	Anticoagulants, 5.6
DM, 21.1		Increased risk, 32.2	Irregularly active, 31.1	Clopidogrel, 0
Dyslipidemia, 82.2	**Obese**, %	OSA diagnosis, 11.1	Active, 14.4	ASA, 6.7
Tobacco abuse, 21.1	25.6		Very active, 3.3	Antilipidemic drugs, 42.2
				Statins, 41.1
				Antihypertensive drugs, 41.1
				Oral antidiabetic drugs, 15.6
				Metformin, 15.6
				Insulin, 4.4
**Diagnosis**, %	**Age of psychiatric disorder onset,** M (SD)	**Psychiatric hospitalizations, M** (SD)	**Family history**, %	**Psychopharmacologic medication, %**
Schizophrenia spectrum and other psychotic disorders, 27.8		2.8 (3.09)	Dementia before 65 years, 7.8	Typical antipsychotics, 10
			Dementia at 65 or more, 24.4	

Bipolar and related disorders, 41.1<br><br>Major Depressive disorder, 31.1	40.16 (14.74); Med – 40<br><br>[14 ; 73]y*	Med - 2	MDD/BP, 38.9<br><br>Schizophrenia/psychosis spectrum disorders, 13.3<br><br>Suicide, 12.2	Atypical antipsychotics, 90<br><br>Lithium, 12.2<br><br>Valproate/valproic acid, 25.6<br><br>Benzodiazepines, 90<br><br>Tricyclic antidepressants, 5.6<br><br>SSRI antidepressants, 43.5<br><br>Other antidepressants, 23.3<br><br>Psychostimulants, 4.4

*Demographic and clinical characteristics of the sample. M – mean, SD – standard deviation, Med – median, BMI – body mass index, ASA – acetylsalicylic acid, OSA – obstructive sleep apnea, MDD – major depressive disorder, BP – bipolar disorder. *Age range of psychiatric disorder onset.*

Supplementary material S3.

Variables	Onset <40y N=44	Onset ≥40y N=46	Stat.(1)	Sig.(2)
**MoCA,** M (SD)	21.52 (4.13)	21.25 (5.21)	U=946[(3)]	0.59
*Visuospatial/executive	3.20 (1.32)	2.69 (1.60)	U=828.5[(3)]	0.13
*Naming	2.84 (0.56)	2.76 (0.73)	U=991[(3)]	0.74
*Attention	4.22 (1.84)	4.32 (1.62)	U=1002[(3)]	0.93
*Language	1.97 (0.90)	2.26 (0.74)	U=852.5[(3)]	0.16
*Abstraction	1.45 (0.79)	1.10 (0.79)	U=762[(3)]	**0.028**
*Memory	1.61 (1.48)	1.52 (1.54)	U=966[(3)]	0.7
*Orientation	5.70 (0.59)	5.71 (0.83)	U=928[(3)]	0.3
**MMSE**, M (SD)	26.70 (2.79)	27.55 (2.24)	U=824[(3)]	0.12
*Orientation	9.15 (1.37)	9.52 (0.93)	U=838[(3)]	0.93
*Registration	3.00 (0)	3.00 (0)	U=1012[(3)]	1
*Attention	4.02 (1.24)	4.17 (1.30)	U=902.5[(3)]	0.33
*Recall	2.20 (0.87)	2.34 (0.60)	U=965[(3)]	0.68

*Language	7.36 (0.83)	7.60 (0.68)	U=844(3)	0.11
*Visual construction	0.77 (0.42)	0.67 (0.47)	U=912(3)	0.29
**FAB**, M (SD)	13.08 (3.74)	12.58 (4.32)	U=592(3)	0.81
**FTT**, M (SD)	46.50 (11.04)	52.36 (7.35)	U=833.5(3)	0.14
**SPES**, M (SD)	2.85 (4.07)	3.27 (4.45)	U=886.5(3)	0.88
**NPI total**, M (SD)	42.43 (18.77)	43.45 (18.50)	T=0.26(1)	0.79
*Delusions	5.84 (5.37)	5.39 (5.45)	U=959(3)	0.64
*Hallucinations	2.13 (4.26)	1.45 (2.70)	U=1010(3)	0.98
*Agitation	4.36 (4.63)	3.71 (4.54)	U=920(3)	0.44
*Depression/dysphoria	3.88 (4.86)	5.04 (5.37)	U=928.5(3)	0.47
*Anxiety	5.11 (4.38)	5.13 (4.29)	U=1004.5(3)	0.95
*Euphoria	2.10 (4.30)	3.36 (4.99)	U=968.5(3)	0.67
*Apathy	2.45 (4.02)	2.02 (4.16)	U=935(3)	0.44
*Disinhibition	1.75 (3.07)	2.21 (3.18)	U=944(3)	0.52
*Irritability	3.31 (4.19)	3.04 (4.28)	U=931.5(3)	0.48
*Motor behavior	2.59 (4.20)	1.65 (2.99)	U=940(3)	0.50
*Nocturnal behavior	6.34 (4.92)	7.56 (4.66)	U=871(3)	0.23
*Appetite	1.93 (3.63)	2.84 (3.99)	U=858(3)	0.14
**SBQ-R**, M (SD)	6.54 (4.14)	6.92 (4.64)	U=1009(3)	0.98
**GAF**, %	3.95 (1.61)	3.82 (1.48)	U=959.5(3)	0.58

*Comparison between individuals with disease onset before age 40 and individuals with disease onset at age 40 or older. (1) Stat. – statistic test, (2) Sig. - significance, (2) Chi-squared test, (3) Mann-Whitney U test, M – mean, SD – standard deviation. MoCA – Montreal Cognitive assessment total score adapted for educational level, MMSE – Mini mental state examination total score, FTT – Finger tapping test, FAB – Frontal assessment battery, SPES – Short Parkinson evaluation scale, NPI – Neuropsychiatric inventory total score, SBQ-R – Suicide Behaviors Questionnaire-Revised, GAF – Global assessment of functioning scale. *Subtest score*

Supplementary material S4.

Variables	Onset <40y<br>N=44	Onset ≥40y<br>N=46	Stat.$^{(1)}$	Sig.
**Fazekas scale**, %			χ^2=1.18$^{(2)}$	0.61
Grade 0	47.7	39.1		
Grade 1	43.2	43.5		
Grade 2	6.8	15.2		
Grade 3	2.3	2.2		
**MSRS**, M (SD)	5.52 (4.55)	5.70 (4.55)	U=1009$^{(3)}$	0.98
*MSRSpv	2.42 (1.33)	2.14 (1.53)	U=988.5$^{(3)}$	0.84
* MSRSpv occipital	0.38 (0.57)	0.47 (0.65)	U=949$^{(3)}$	0.54
* MSRSpv frontal	1.11 (0.49)	1.13 (0.58)	U=991.5$^{(3)}$	0.83
* MSRSpv lateral walls	0.93 (0.50)	0.89 (0.60)	U=972$^{(3)}$	0.69
*MSRSd	3.09 (3.54)	2.08 (3.83)	U=986$^{(3)}$	0.83
* MSRSd frontal	1.54 (1.78)	1.73 (1.85)	U=945$^{(3)}$	0.57
* MSRSd parietal	1.43 (1.93)	1.43 (1.86)	U=1000$^{(3)}$	0.91
* MSRSd occipital	0.02 (0.15)	0.08 (0.46)	U=990.5$^{(3)}$	0.57
* MSRSd temporal	0.09 (0.29)	0.15 (0.41)	U=970$^{(3)}$	0.53
**MTA**, M (SD)	0.63 (0.68)	0.51 (0.63)	U=816$^{(3)}$	0.39
**Koedam score**, M (SD)	0.47 (0.66)	0.53 (0.59)	U=986$^{(3)}$	0.80
**GCA-F**, M (SD)	0.54 (0.79)	0.60 (0.66)	U=907$^{(3)}$	0.34

*Comparison between individuals with disease onset before age 40 and individuals with disease onset at age 40 or older. (1) Stat. – statistic test, Sig. – significance (2) Chi-squared test, (3) Mann-Whitney U test, M – mean, SD – standard deviation. MSRS - Modified Scheltens rating scale, MSRSpv – periventricular lesions subscore, MSRSd – deep lesions subscore, MTA – Medial temporal atrophy scale, GCA-F – Global cortical atrophy/Pasquier scale frontal subscale. *Subtest score*

Supplementary material S5.

	Onset ≥40 years, N=46			
	MSRS PV		MSRSd	
**Variables**	**Stat. (τ)**	**Sig.**	**Stat. (τ)**	**Sig.**
**MoCA**	-.267	0.073	-0.236	0.113
*Visuospatial/executive	-0.195	0.193	-0.140	0.351
*Naming	-0.046	0.759	-0.081	0.589
*Attention	0.064	0.671	0.119	0.429
*Language	-0.126	0.401	-0.038	0.797
*Abstraction	-0.249	0.094	-0.246	0.098
*Memory	-0.143	0.342	-0.251	0.091
*Orientation	-0.402	**0.005**	-0.354	**0.015**
**MMSE**	-0.113	0.450	-0.089	0.554
*Orientation	-0.165	0.271	-0.144	0.337
*Registration	.	.	.	.
*Attention	0.090	0.547	0.072	0.630
*Recall	0.068	0.649	-0.081	0.589
*Language	0	0.999	0.109	0.469
*Visual construction	-0.107	0.476	-0.222	0.137
**FAB**	0.035	0.836	-0.059	0.732
**FFT**	-0.046	0.756	-0.159	0.289
**SPES**	0.391	**0.008**	0.419	**0.004**
**Psychiatric hospitalizations**	0	0.998	0.056	0.707

**NPI total**	-0.196	0.191	-0.315	**0.032**
*Delusions	-0.318	**0.031**	-0.348	**0.017**
*Hallucinations	-0.203	0.176	-0.110	0.463
*Agitation	0.003	0.982	-0.243	0.102
*Depression/dysphoria	-0.105	0.486	0.019	0.896
*Anxiety	-0.038	0.798	-0.131	0.384
*Euphoria	0.142	0.345	-0.114	0.449
*Apathy	-0.128	0.393	0.107	0.477
*Disinhibition	0.078	0.601	-0.163	0.278
*Irritability	-0.071	0.634	-0.362	**0.013**
*Motor behavior	0.187	0.212	0.142	0.344
*Nocturnal behavior	-0.076	0.614	-0.084	0.577
*Appetite	-0.181	0.227	-0.007	0.957
**SBQ-R**	0.141	0.349	0.333	**0.023**
**MTA**	0.204	0.198	0.117	0.462
**Koedam score**	-0.160	0.287	0.041	0.783
**GCA-F**	0.175	0.244	0.265	0.074
**Body mass index**	-0.063	0.675	-0.083	0.582
**Glycate hemoglobin**	0.318	**0.0395**	0.254	0.103

Kendall's tau correlation analysis between multiple variables of the subgroup of patients with late onset. τ- Kendall's tau correlation coefficient, MSRS - Modified Scheltens rating scale, MSRS PV - Modified Scheltens rating scale periventricular lesions, MSRSd - Modified Scheltens rating scale deep lesions, MoCA – Montreal Cognitive assessment total score, MMSE – Mini mental state examination total score, FTT – Finger tapping test, FAB – Frontal assessment battery, SPES – Short Parkinson evaluation scale, NPI – Neuropsychiatric inventory total score, SBQ-R – Suicide Behaviors Questionnaire-Revised, MTA – Medial temporal atrophy scale, GCA-F – Global cortical atrophy/Pasquier scale frontal subscale.

Supplementary material S6.

	Onset ≥40 years, N=46							
	**MSRSd frontal**		**MSRSd parietal**		**MSRSd occipital**		**MSRSd temporal**	
**Variables**	**Stat. (τ)**	**Sig.**	**Stat. (τ)**	**Sig.**	**Stat. (τ)**	**Sig.**	**Stat. (τ)**	**Sig.**
**MoCA**	-0.252	0.090	-0.184	0.218	-0.204	0.172	-0.117	0.437
*Visuospatial/executive	-0.173	0.247	-0.071	0.638	-0.021	0.886	-0.065	0.667
*Naming	-0.111	0.460	-0.053	0.724	-0.244	0.101	0.134	0.371
*Attention	0.111	0.458	0.085	0.571	-0.247	0.096	-0.018	0.901
*Language	-0.101	0.502	0.071	0.636	0.074	0.620	0.016	0.911
*Abstraction	-0.287	0.052	-0.087	0.561	-0.290	0.050	-0.224	0.134
*Memory	-0.193	0.196	-0.271	0.067	-0.062	0.678	-0.110	0.465
*Orientation	-0.327	**0.026**	-0.386	**0.007**	-0.199	0.183	-0.419	**0.003**
**MMSE**	-0.065	0.664	-0.124	0.411	-0.147	0.327	-0.168	0.263
*Orientation	-0.167	0.267	-0.146	0.331	-0.298	0.413	-0.298	**0.044**
*Registration	.	.	.	.	.	.	.	.
*Attention	0.020	0.890	0.155	0.301	-0.068	0.995	-0.068	0.652
*Recall	-0.009	0.947	-0.156	0.299	0.025	0.721	0.025	0.865
*Language	0.081	0.588	0.159	0.290	0.099	0.325	0.099	0.508
*Visual construction	-0.191	0.203	-0.192	0.199	-0.152	0.578	-0.152	0.311
**FAB**	-0.095	0.578	-0.035	0.838	-0.041	0.811	0.109	0.525
**FFT**	-0.196	0.191	-0.112	0.456	-0.107	0.478	-0.032	0.827
**SPES**	0.421	**0.004**	0.428	**0.003**	0.029	0.850	0.448	**0.002**
**Psychiatric hospitalizations**	0	0.996	0.077	0.608	0.047	0.756	0.328	**0.025**

**NPI total**	-0.243	0.102	-0.431	**0.002**	-0.111	0.460	-0.140	0.352
*Delusions	-0.352	**0.016**	-0.310	**0.035**	0.012	0.935	-0.080	0.596
*Hallucinations	-0.084	0.575	-0.104	0.488	0.126	0.400	-0.130	0.385
*Agitation	-0.154	0.304	-0.336	**0.022**	-0.093	0.536	-0.122	0.416
*Depression/dysphoria	0.012	0.932	-0.007	0.962	0.020	0.891	0.057	0.704
*Anxiety	-0.174	0.246	-0.154	0.304	0.179	0.231	0.034	0.820
*Euphoria	-0.071	0.637	-0.142	0.345	-0.151	0.313	0.022	0.884
*Apathy	0.136	0.363	0.076	0.614	-0.124	0.407	-0.226	0.129
*Disinhibition	-0.119	0.428	-0.204	0.171	-0.158	0.292	-0.028	0.851
*Irritability	-0.324	**0.027**	-0.331	**0.024**	-0.171	0.254	-0.228	0.126
*Motor behavior	0.221	0.139	-0.006	0.964	0.001	0.992	0.030	0.841
*Nocturnal behavior	-0.024	0.872	-0.190	0.205	0.084	0.574	-0.093	0.534
*Appetite	0.036	0.808	-0.139	0.355	-0.177	0.237	-0.064	0.668
**SBQ-R**	0.250	0.093	0.313	**0.033**	0.251	0.092	0.341	**0.020**
**MTA**	0.117	0.462	0.098	0.539	0.005	0.971	0.276	0.080
**Koedam score**	0.025	0.864	0.096	0.523	0.022	0.880	-0.078	0.604
**GCA-F**	0.211	0.157	0.286	0.053	-0.027	0.858	0.129	0.389
**Body mass index**	-0.217	0.146	0.080	0.594	-0.082	0.585	0.043	0.772
**Glycate hemoglobin**	0.285	0.066	0.125	0.427	0.257	0.099	0.286	0.066

Kendall's tau correlation analysis between multiple variables of the subgroup of patients with late onset. τ- Kendall's tau correlation coefficient, MSRS - Modified Scheltens rating scale, MSRS PV - Modified Scheltens rating scale periventricular lesions, MSRSd - Modified Scheltens rating scale deep lesions, MoCA – Montreal Cognitive assessment total score, MMSE – Mini mental state examination total score, FTT – Finger tapping test, FAB – Frontal assessment battery, SPES – Short Parkinson evaluation scale, NPI – Neuropsychiatric inventory total score, SBQ-R – Suicide Behaviors Questionnaire-Revised, MTA – Medial temporal atrophy scale, GCA-F – Global cortical atrophy/Pasquier scale frontal subscale.

Supplementary material S7.

	Onset ≥40 years, N=46					
	MSRSpv occipital		MRSRpv frontal		MRSR lateral walls	
**Variables**	**Stat. (τ)**	**Sig.**	**Stat. (τ)**	**Sig.**	**Stat. (τ)**	**Sig.**
**MoCA**	-0.245	0.099	-0.190	0.205	-0.227	0.128
*Visuospatial/executive	-0.280	0.059	-0.050	0.737	-0.103	0.492
*Naming	0.021	0.889	-0.023	0.878	-0.076	0.612
*Attention	0.092	0.541	-0.029	0.846	0.046	0.757
*Language	-0.050	0.738	-0.074	0.620	-0.097	0.520
*Abstraction	-0.059	0.693	-0.276	0.062	-0.295	**0.046**
*Memory	-0.241	0.105	0.041	0.784	-0.107	0.475
*Orientation	-0.313	**0.033**	-0.371	**0.011**	-0.384	**0.008**
**MMSE**	-0.021	0.885	-0.158	0.293	-0.104	0.489
*Orientation	0.016	0.912	-0.253	0.089	-0.279	0.059
*Registration	.	.	.	.	.	.
*Attention	0.238	0.111	-0.091	0.546	0.018	0.900
*Recall	-0.026	0.862	0.130	0.388	0.114	0.449
*Language	0.014	0.923	-0.050	0.739	0.025	0.866
*Visual construction	-0.169	0.259	0.004	0.978	0.020	0.476
**FAB**	-0.071	0.680	0.210	0.217	0.060	0.726
**FFT**	-0.025	0.864	-0.089	0.552	-0.073	0.625
**SPES**	0.377	**0.011**	0.241	0.114	0.308	**0.041**
**Psychiatric hospitalizations**	-0.102	0.499	0.065	0.667	0.021	0.889

**NPI total**	-0.238	0.111	-0.088	0.559	-0.085	0.574
*Delusions	-0.386	**0.008**	-0.131	0.384	-0.211	0.158
*Hallucinations	-0.130	0.388	-0.003	0.981	-0.265	0.074
*Agitation	-0.140	0.350	0.106	0.482	0.083	0.580
*Depression/dysphoria	-0.018	0.901	-0.085	0.571	-0.126	0.400
*Anxiety	-0.021	0.889	0.026	0.858	-0.004	0.975
*Euphoria	0.060	0.688	0.080	0.596	0.213	0.155
*Apathy	-0.050	0.737	-0.191	0.201	-0.117	0.436
*Disinhibition	0.036	0.810	-0.025	0.868	0.170	0.258
*Irritability	-0.088	0.560	-0.111	0.460	0.038	0.800
*Motor behavior	-0.040	0.788	0.226	0.130	0.261	0.079
*Nocturnal behavior	-0.078	0.605	-0.061	0.683	-0.019	0.896
*Appetite	-0.265	0.074	-0.088	0.556	-0.124	0.408
**SBQ-R**	0.156	0.298	0.231	0.121	0.027	0.855
**MTA**	0.145	0.363	0.124	0.438	0.105	0.509
**Koedam score**	-0.159	0.290	0.052	0.730	-0.288	0.052
**GCA-F**	0.157	0.294	0.079	0.598	0.026	0.861
**Body mass index**	0.044	0.770	-0.156	0.299	-0.053	0.721
**Glycate hemoglobin**	0.216	0.167	0.451	**0.002**	0.247	0.114

Kendall's tau correlation analysis between multiple variables of the subgroup of patients with late onset. τ- Kendall's tau correlation coefficient, MSRS - Modified Scheltens rating scale, MSRSpv - Modified Scheltens rating scale periventricular lesions, MSRSd - Modified Scheltens rating scale deep lesions, MoCA – Montreal Cognitive assessment total score, MMSE – Mini mental state examination total score, FTT – Finger tapping test, FAB – Frontal assessment battery, SPES – Short Parkinson evaluation scale, NPI – Neuropsychiatric inventory total score, SBQ-R – Suicide Behaviors Questionnaire-Revised, MTA – Medial temporal atrophy scale, GCA-F – Global cortical atrophy/Pasquier scale frontal subscale.

Supplementary material S8.

Benjamini–Hochberg FDR (False Discovery Rate) correction applied within families of tests defined by categories (anthropometric cognition, neuropsychiatric, motor, neuroimaging, metabolic/vascular). The significance level adopted was p<0.05 after correction.

WMH_metric	Variable	tau	Sig.	Category	Sig.
Fazekas	Body mass index	-0,005	0,971	Anthropometric	0,971
MSRS_PV_frontal	Body mass index	-0,156	0,299	Anthropometric	0,299
MSRS_PV_lateral	Body mass index	-0,053	0,721	Anthropometric	0,721
MSRS_PV_occipital	Body mass index	0,044	0,77	Anthropometric	0,77
MSRS_PV_total	Body mass index	-0,063	0,675	Anthropometric	0,675
MSRS_deep_frontal	Body mass index	-0,217	0,146	Anthropometric	0,146
MSRS_deep_occipital	Body mass index	-0,082	0,585	Anthropometric	0,585
MSRS_deep_parietal	Body mass index	0,08	0,594	Anthropometric	0,594
MSRS_deep_temporal	Body mass index	0,043	0,772	Anthropometric	0,772
MSRS_deep_total	Body mass index	-0,083	0,582	Anthropometric	0,582
MSRS_total	Body mass index	-0,056	0,711	Anthropometric	0,711

Benjamini–Hochberg FDR (False Discovery Rate) correction applied within families of tests defined by categories - anthropometric category. The significance level adopted was p<0.05 after correction (Sig.).

WMH_metric	Variable	tau	Sig.	Category	Sig.
Fazekas	GCA-F	0,39	0,007	Atrophy/Neuroimaging	**0,021**
Fazekas	Koedam score	-0,089	0,554	Atrophy/Neuroimaging	0,554

Fazekas	MTA	0,182	0,253	Atrophy/Neuroimaging	0,3795
MSRS_PV_frontal	GCA-F	0,079	0,598	Atrophy/Neuroimaging	0,73
MSRS_PV_frontal	Koedam score	0,052	0,73	Atrophy/Neuroimaging	0,73
MSRS_PV_frontal	MTA	0,124	0,438	Atrophy/Neuroimaging	0,73
MSRS_PV_lateral	GCA-F	0,026	0,861	Atrophy/Neuroimaging	0,861
MSRS_PV_lateral	Koedam score	-0,288	0,052	Atrophy/Neuroimaging	0,156
MSRS_PV_lateral	MTA	0,105	0,509	Atrophy/Neuroimaging	0,7635
MSRS_PV_occipital	GCA-F	0,157	0,294	Atrophy/Neuroimaging	0,363
MSRS_PV_occipital	Koedam score	-0,159	0,29	Atrophy/Neuroimaging	0,363
MSRS_PV_occipital	MTA	0,145	0,363	Atrophy/Neuroimaging	0,363
MSRS_PV_total	GCA-F	0,175	0,244	Atrophy/Neuroimaging	0,287
MSRS_PV_total	Koedam score	-0,16	0,287	Atrophy/Neuroimaging	0,287
MSRS_PV_total	MTA	0,204	0,198	Atrophy/Neuroimaging	0,287
MSRS_deep_frontal	GCA-F	0,211	0,157	Atrophy/Neuroimaging	0,471
MSRS_deep_frontal	Koedam score	0,025	0,864	Atrophy/Neuroimaging	0,864
MSRS_deep_frontal	MTA	0,117	0,462	Atrophy/Neuroimaging	0,693
MSRS_deep_occipital	GCA-F	-0,027	0,858	Atrophy/Neuroimaging	0,971
MSRS_deep_occipital	Koedam score	0,022	0,88	Atrophy/Neuroimaging	0,971
MSRS_deep_occipital	MTA	0,005	0,971	Atrophy/Neuroimaging	0,971
MSRS_deep_parietal	GCA-F	0,286	0,053	Atrophy/Neuroimaging	0,159
MSRS_deep_parietal	Koedam score	0,096	0,523	Atrophy/Neuroimaging	0,539
MSRS_deep_parietal	MTA	0,098	0,539	Atrophy/Neuroimaging	0,539
MSRS_deep_temporal	GCA-F	0,129	0,389	Atrophy/Neuroimaging	0,5835
MSRS_deep_temporal	Koedam score	-0,078	0,604	Atrophy/Neuroimaging	0,604

MSRS_deep_temporal	MTA	0,276	0,08	Atrophy/Neuroimaging	0,24
MSRS_deep_total	GCA-F	0,265	0,074	Atrophy/Neuroimaging	0,222
MSRS_deep_total	Koedam score	0,041	0,783	Atrophy/Neuroimaging	0,783
MSRS_deep_total	MTA	0,117	0,462	Atrophy/Neuroimaging	0,693
MSRS_total	GCA-F	0,248	0,095	Atrophy/Neuroimaging	0,285
MSRS_total	Koedam score	-0,053	0,724	Atrophy/Neuroimaging	0,724
MSRS_total	MTA	0,139	0,383	Atrophy/Neuroimaging	0,5745

Benjamini–Hochberg FDR (False Discovery Rate) correction applied within families of tests defined by categories - Atrophy/Neuroimaging category. The significance level adopted was p<0.05 after correction (Sig.).

**WMH_metric**	**Variable**	**tau**	**Sig.**	**Category**	**Sig.**
Fazekas	Psychiatric hospitalizations	-0,087	0,561	Clinical	0,561
MSRS_PV_frontal	Psychiatric hospitalizations	0,065	0,667	Clinical	0,667
MSRS_PV_lateral	Psychiatric hospitalizations	0,021	0,889	Clinical	0,889
MSRS_PV_occipital	Psychiatric hospitalizations	-0,102	0,499	Clinical	0,499
MSRS_PV_total	Psychiatric hospitalizations	0	0,998	Clinical	0,998
MSRS_deep_frontal	Psychiatric hospitalizations	0	0,996	Clinical	0,996
MSRS_deep_occipital	Psychiatric hospitalizations	0,047	0,756	Clinical	0,756
MSRS_deep_parietal	Psychiatric hospitalizations	0,077	0,608	Clinical	0,608
MSRS_deep_temporal	Psychiatric hospitalizations	0,328	0,025	Clinical	**0,025**
MSRS_deep_total	Psychiatric hospitalizations	0,056	0,707	Clinical	0,707
MSRS_total	Psychiatric hospitalizations	0,029	0,849	Clinical	0,849

Benjamini–Hochberg FDR (False Discovery Rate) correction applied within families of tests defined by categories - Clinical category. The significance level adopted was p<0.05 after correction (Sig.).

WMH_metric	Variable	tau	Sig.	Category	Sig.
Fazekas	Abstraction	-0,358	0,014	Cognitive	**0,049**
Fazekas	Attention	-0,03	0,838	Cognitive	0,942
Fazekas	Attention	-0,01	0,645	Cognitive	0,82090909
Fazekas	FAB	-0,159	0,353	Cognitive	0,4942
Fazekas	Language	-0,197	0,187	Cognitive	0,29088889
Fazekas	Language	-0,2	0,942	Cognitive	0,942
Fazekas	Memory	-0,369	0,011	Cognitive	**0,049**
Fazekas	MoCA.	-0,437	0,002	Cognitive	**0,028**
Fazekas	Naming	-0,211	0,157	Cognitive	0,27475
Fazekas	Orientation	-0,359	0,014	Cognitive	**0,049**
Fazekas	Orientation	-0,313	0,063	Cognitive	0,126
Fazekas	Recall	-0,011	0,942	Cognitive	0,942
Fazekas	Registration	-0,06	0,033	Cognitive	0,08166667
Fazekas	Visuospatial/executive	-0,311	0,035	Cognitive	0,08166667
MSRS_PV_frontal	Abstraction	-0,276	0,062	Cognitive	0,41533333
MSRS_PV_frontal	Attention	-0,029	0,846	Cognitive	0,878
MSRS_PV_frontal	Attention	0,13	0,546	Cognitive	0,878
MSRS_PV_frontal	FAB	0,21	0,217	Cognitive	0,6076

MSRS_PV_frontal	Language	-0,074	0,62	Cognitive	0,878
MSRS_PV_frontal	Language	0,004	0,739	Cognitive	0,878
MSRS_PV_frontal	Memory	0,041	0,784	Cognitive	0,878
MSRS_PV_frontal	MoCA.	-0,19	0,205	Cognitive	0,6076
MSRS_PV_frontal	Naming	-0,023	0,878	Cognitive	0,878
MSRS_PV_frontal	Orientation	-0,371	0,011	Cognitive	0,154
MSRS_PV_frontal	Orientation	-0,253	0,293	Cognitive	0,68366667
MSRS_PV_frontal	Recall	-0,05	0,388	Cognitive	0,776
MSRS_PV_frontal	Registration	-0,091	0,089	Cognitive	0,41533333
MSRS_PV_frontal	Visuospatial/executive	-0,05	0,737	Cognitive	0,878
MSRS_PV_lateral	Abstraction	-0,295	0,046	Cognitive	0,27533333
MSRS_PV_lateral	Attention	0,046	0,757	Cognitive	0,88316667
MSRS_PV_lateral	Attention	0,114	0,9	Cognitive	0,9
MSRS_PV_lateral	FAB	0,06	0,726	Cognitive	0,88316667
MSRS_PV_lateral	Language	-0,097	0,52	Cognitive	0,80888889
MSRS_PV_lateral	Language	0,02	0,866	Cognitive	0,9
MSRS_PV_lateral	Memory	-0,107	0,475	Cognitive	0,80888889
MSRS_PV_lateral	MoCA.	-0,227	0,128	Cognitive	0,448
MSRS_PV_lateral	Naming	-0,076	0,612	Cognitive	0,8568
MSRS_PV_lateral	Orientation	-0,384	0,008	Cognitive	0,112
MSRS_PV_lateral	Orientation	-0,279	0,489	Cognitive	0,80888889
MSRS_PV_lateral	Recall	0,025	0,449	Cognitive	0,80888889
MSRS_PV_lateral	Registration	0,018	0,059	Cognitive	0,27533333

MSRS_PV_lateral	Visuospatial/executive	-0,103	0,492	Cognitive	0,80888889
MSRS_PV_occipital	Abstraction	-0,059	0,693	Cognitive	0,923
MSRS_PV_occipital	Attention	0,092	0,541	Cognitive	0,923
MSRS_PV_occipital	Attention	-0,026	0,111	Cognitive	0,3108
MSRS_PV_occipital	FAB	-0,071	0,68	Cognitive	0,923
MSRS_PV_occipital	Language	-0,05	0,738	Cognitive	0,923
MSRS_PV_occipital	Language	-0,169	0,923	Cognitive	0,923
MSRS_PV_occipital	Memory	-0,241	0,105	Cognitive	0,3108
MSRS_PV_occipital	MoCA.	-0,245	0,099	Cognitive	0,3108
MSRS_PV_occipital	Naming	0,021	0,889	Cognitive	0,923
MSRS_PV_occipital	Orientation	-0,313	0,033	Cognitive	0,3108
MSRS_PV_occipital	Orientation	0,016	0,885	Cognitive	0,923
MSRS_PV_occipital	Recall	0,014	0,862	Cognitive	0,923
MSRS_PV_occipital	Registration	0,238	0,912	Cognitive	0,923
MSRS_PV_occipital	Visuospatial/executive	-0,28	0,059	Cognitive	0,3108
MSRS_PV_total	Abstraction	-0,249	0,094	Cognitive	0,43866667
MSRS_PV_total	Attention	0,064	0,671	Cognitive	0,854
MSRS_PV_total	Attention	0,068	0,547	Cognitive	0,85088889
MSRS_PV_total	FAB	0,035	0,836	Cognitive	0,90030769
MSRS_PV_total	Language	-0,126	0,401	Cognitive	0,7875
MSRS_PV_total	Language	-0,107	0,999	Cognitive	0,999
MSRS_PV_total	Memory	-0,143	0,342	Cognitive	0,7875
MSRS_PV_total	MoCA.	-0,267	0,073	Cognitive	0,43866667

MSRS_PV_total	Naming	-0,046	0,759	Cognitive	0,8855
MSRS_PV_total	Orientation	-0,402	0,005	Cognitive	0,07
MSRS_PV_total	Orientation	-0,165	0,45	Cognitive	0,7875
MSRS_PV_total	Recall	0	0,649	Cognitive	0,854
MSRS_PV_total	Registration	0,09	0,271	Cognitive	0,7588
MSRS_PV_total	Visuospatial/executive	-0,195	0,193	Cognitive	0,6755
MSRS_deep_frontal	Abstraction	-0,287	0,052	Cognitive	0,364
MSRS_deep_frontal	Attention	0,111	0,458	Cognitive	0,74836364
MSRS_deep_frontal	Attention	-0,009	0,89	Cognitive	0,947
MSRS_deep_frontal	FAB	-0,095	0,578	Cognitive	0,74836364
MSRS_deep_frontal	Language	-0,101	0,502	Cognitive	0,74836364
MSRS_deep_frontal	Language	-0,191	0,588	Cognitive	0,74836364
MSRS_deep_frontal	Memory	-0,193	0,196	Cognitive	0,623
MSRS_deep_frontal	MoCA.	-0,252	0,09	Cognitive	0,42
MSRS_deep_frontal	Naming	-0,111	0,46	Cognitive	0,74836364
MSRS_deep_frontal	Orientation	-0,327	0,026	Cognitive	0,364
MSRS_deep_frontal	Orientation	-0,167	0,664	Cognitive	0,77466667
MSRS_deep_frontal	Recall	0,081	0,947	Cognitive	0,947
MSRS_deep_frontal	Registration	0,02	0,267	Cognitive	0,623
MSRS_deep_frontal	Visuospatial/executive	-0,173	0,247	Cognitive	0,623
MSRS_deep_occipital	Abstraction	-0,29	0,05	Cognitive	0,47133333
MSRS_deep_occipital	Attention	-0,247	0,096	Cognitive	0,47133333

MSRS_deep_occipital	Attention	0,025	0,995	Cognitive	0,995
MSRS_deep_occipital	FAB	-0,041	0,811	Cognitive	0,94616667
MSRS_deep_occipital	Language	0,074	0,62	Cognitive	0,91763636
MSRS_deep_occipital	Language	-0,152	0,325	Cognitive	0,654
MSRS_deep_occipital	Memory	-0,062	0,678	Cognitive	0,91763636
MSRS_deep_occipital	MoCA.	-0,204	0,172	Cognitive	0,5124
MSRS_deep_occipital	Naming	-0,244	0,101	Cognitive	0,47133333
MSRS_deep_occipital	Orientation	-0,199	0,183	Cognitive	0,5124
MSRS_deep_occipital	Orientation	-0,298	0,327	Cognitive	0,654
MSRS_deep_occipital	Recall	0,099	0,721	Cognitive	0,91763636
MSRS_deep_occipital	Registration	-0,068	0,413	Cognitive	0,72275
MSRS_deep_occipital	Visuospatial/executive	-0,021	0,886	Cognitive	0,95415385
MSRS_deep_parietal	Abstraction	-0,087	0,561	Cognitive	0,74433333
MSRS_deep_parietal	Attention	0,085	0,571	Cognitive	0,74433333
MSRS_deep_parietal	Attention	-0,156	0,301	Cognitive	0,662
MSRS_deep_parietal	FAB	-0,035	0,838	Cognitive	0,838
MSRS_deep_parietal	Language	0,071	0,636	Cognitive	0,74433333
MSRS_deep_parietal	Language	-0,192	0,29	Cognitive	0,662
MSRS_deep_parietal	Memory	-0,271	0,067	Cognitive	0,469
MSRS_deep_parietal	MoCA.	-0,184	0,218	Cognitive	0,662
MSRS_deep_parietal	Naming	-0,053	0,724	Cognitive	0,77969231
MSRS_deep_parietal	Orientation	-0,386	0,007	Cognitive	0,098

MSRS_deep_parietal	Orientation	-0,146	0,411	Cognitive	0,71925
MSRS_deep_parietal	Recall	0,159	0,299	Cognitive	0,662
MSRS_deep_parietal	Registration	0,155	0,331	Cognitive	0,662
MSRS_deep_parietal	Visuospatial/executive	-0,071	0,638	Cognitive	0,74433333
MSRS_deep_temporal	Abstraction	-0,224	0,134	Cognitive	0,62533333
MSRS_deep_temporal	Attention	-0,018	0,901	Cognitive	0,911
MSRS_deep_temporal	Attention	0,025	0,652	Cognitive	0,84890909
MSRS_deep_temporal	FAB	0,109	0,525	Cognitive	0,81666667
MSRS_deep_temporal	Language	0,016	0,911	Cognitive	0,911
MSRS_deep_temporal	Language	-0,152	0,508	Cognitive	0,81666667
MSRS_deep_temporal	Memory	-0,11	0,465	Cognitive	0,81666667
MSRS_deep_temporal	MoCA.	-0,117	0,437	Cognitive	0,81666667
MSRS_deep_temporal	Naming	0,134	0,371	Cognitive	0,81666667
MSRS_deep_temporal	Orientation	-0,419	0,003	Cognitive	**0,042**
MSRS_deep_temporal	Orientation	-0,298	0,263	Cognitive	0,81666667
MSRS_deep_temporal	Recall	0,099	0,865	Cognitive	0,911
MSRS_deep_temporal	Registration	-0,068	0,044	Cognitive	0,308
MSRS_deep_temporal	Visuospatial/executive	-0,065	0,667	Cognitive	0,84890909
MSRS_deep_total	Abstraction	-0,246	0,098	Cognitive	0,3955
MSRS_deep_total	Attention	0,119	0,429	Cognitive	0,735
MSRS_deep_total	Attention	-0,081	0,63	Cognitive	0,735
MSRS_deep_total	FAB	-0,059	0,732	Cognitive	0,78830769

MSRS_deep_total	Language	-0,038	0,797	Cognitive	0,797
MSRS_deep_total	Language	-0,222	0,469	Cognitive	0,735
MSRS_deep_total	Memory	-0,251	0,091	Cognitive	0,3955
MSRS_deep_total	MoCA.	-0,236	0,113	Cognitive	0,3955
MSRS_deep_total	Naming	-0,081	0,589	Cognitive	0,735
MSRS_deep_total	Orientation	-0,354	0,015	Cognitive	0,21
MSRS_deep_total	Orientation	-0,144	0,554	Cognitive	0,735
MSRS_deep_total	Recall	0,109	0,589	Cognitive	0,735
MSRS_deep_total	Registration	0,072	0,337	Cognitive	0,735
MSRS_deep_total	Visuospatial/executive	-0,14	0,351	Cognitive	0,735
MSRS_total	Abstraction	-0,257	0,084	Cognitive	0,294
MSRS_total	Attention	0,12	0,426	Cognitive	0,69183333
MSRS_total	Attention	-0,04	0,535	Cognitive	0,69183333
MSRS_total	FAB	-0,017	0,92	Cognitive	0,92
MSRS_total	Language	-0,094	0,532	Cognitive	0,69183333
MSRS_total	Language	-0,183	0,593	Cognitive	0,69183333
MSRS_total	Memory	-0,258	0,082	Cognitive	0,294
MSRS_total	MoCA	-0,259	0,081	Cognitive	0,294
MSRS_total	Naming	-0,085	0,572	Cognitive	0,69183333
MSRS_total	Orientation	-0,378	0,009	Cognitive	0,126
MSRS_total	Orientation	-0,162	0,518	Cognitive	0,69183333
MSRS_total	Recall	0,08	0,787	Cognitive	0,84753846

MSRS_total	Registration	0,093	0,28	Cognitive	0,65333333
MSRS_total	Visuospatial/executive	-<br>0,166	0,267	Cognitive	0,65333333

Benjamini–Hochberg FDR (False Discovery Rate) correction applied within families of tests defined by categories - Cognitive category. The significance level adopted was p<0.05 after correction (Sig.).

**WMH_metric**	**Variable**	**tau**	**Sig.**	**Category**	**Sig.**
Fazekas	Folic acid levels	0,348	0,017	Metabolic/vascular	0,136
Fazekas	Glycate hemoglobin	0,289	0,063	Metabolic/vascular	0,2
Fazekas	HDL cholesterol	-0,018	0,903	Metabolic/vascular	0,903
Fazekas	LDL cholesterol	0,033	0,826	Metabolic/vascular	0,903
Fazekas	TGC	0,206	0,168	Metabolic/vascular	0,336
Fazekas	Total cholesterol	0,037	0,802	Metabolic/vascular	0,903
Fazekas	Vitamin B12	0,264	0,075	Metabolic/vascular	0,2
Fazekas	Vitamin D	-0,04	0,786	Metabolic/vascular	0,903
MSRS_PV_frontal	Folic acid levels	0,17	0,256	Metabolic/vascular	0,68266667
MSRS_PV_frontal	Glycate hemoglobin	0,451	0,002	Metabolic/vascular	**0,016**
MSRS_PV_frontal	HDL cholesterol	0,012	0,932	Metabolic/vascular	0,932
MSRS_PV_frontal	LDL cholesterol	-0,024	0,873	Metabolic/vascular	0,932
MSRS_PV_frontal	TGC	0,194	0,195	Metabolic/vascular	0,68266667
MSRS_PV_frontal	Total cholesterol	0,024	0,869	Metabolic/vascular	0,932
MSRS_PV_frontal	Vitamin B12	0,079	0,6	Metabolic/vascular	0,932
MSRS_PV_frontal	Vitamin D	-0,061	0,701	Metabolic/vascular	0,932

MSRS_PV_lateral	Folic acid levels	0,393	0,006	Metabolic/vascular	**0,048**
MSRS_PV_lateral	Glycate hemoglobin	0,247	0,114	Metabolic/vascular	0,304
MSRS_PV_lateral	HDL cholesterol	-0,127	0,042	Metabolic/vascular	0,168
MSRS_PV_lateral	LDL cholesterol	-0,095	0,528	Metabolic/vascular	0,561
MSRS_PV_lateral	TGC	0,12	0,425	Metabolic/vascular	0,561
MSRS_PV_lateral	Total cholesterol	-0,087	0,561	Metabolic/vascular	0,561
MSRS_PV_lateral	Vitamin B12	0,208	0,164	Metabolic/vascular	0,328
MSRS_PV_lateral	Vitamin D	0,097	0,54	Metabolic/vascular	0,561
MSRS_PV_occipital	Folic acid levels	0	0,996	Metabolic/vascular	0,996
MSRS_PV_occipital	Glycate hemoglobin	0,216	0,167	Metabolic/vascular	0,996
MSRS_PV_occipital	HDL cholesterol	0,02	0,891	Metabolic/vascular	0,996
MSRS_PV_occipital	LDL cholesterol	-0,009	0,95	Metabolic/vascular	0,996
MSRS_PV_occipital	TGC	0,022	0,883	Metabolic/vascular	0,996
MSRS_PV_occipital	Total cholesterol	-0,012	0,933	Metabolic/vascular	0,996
MSRS_PV_occipital	Vitamin B12	0,019	0,895	Metabolic/vascular	0,996
MSRS_PV_occipital	Vitamin D	-0,172	0,274	Metabolic/vascular	0,996
MSRS_PV_total	Folic acid levels	0,286	0,053	Metabolic/vascular	0,212
MSRS_PV_total	Glycate hemoglobin	0,318	0,0395	Metabolic/vascular	0,212
MSRS_PV_total	HDL cholesterol	-0,042	0,777	Metabolic/vascular	0,788
MSRS_PV_total	LDL cholesterol	-0,08	0,592	Metabolic/vascular	0,788
MSRS_PV_total	TGC	0,092	0,541	Metabolic/vascular	0,788
MSRS_PV_total	Total cholesterol	-0,067	0,657	Metabolic/vascular	0,788

MSRS_PV_total	Vitamin B12	0,097	0,521	Metabolic/vascular	0,788
MSRS_PV_total	Vitamin D	-0,042	0,788	Metabolic/vascular	0,788
MSRS_deep_frontal	Folic acid levels	0,133	0,374	Metabolic/vascular	0,854
MSRS_deep_frontal	Glycate hemoglobin	0,285	0,066	Metabolic/vascular	0,528
MSRS_deep_frontal	HDL cholesterol	0,095	0,526	Metabolic/vascular	0,854
MSRS_deep_frontal	LDL cholesterol	0,042	0,779	Metabolic/vascular	0,854
MSRS_deep_frontal	TGC	0,082	0,587	Metabolic/vascular	0,854
MSRS_deep_frontal	Total cholesterol	0,064	0,669	Metabolic/vascular	0,854
MSRS_deep_frontal	Vitamin B12	0,028	0,851	Metabolic/vascular	0,854
MSRS_deep_frontal	Vitamin D	0,029	0,854	Metabolic/vascular	0,854
MSRS_deep_occipital	Folic acid levels	0,097	0,518	Metabolic/vascular	0,592
MSRS_deep_occipital	Glycate hemoglobin	0,257	0,099	Metabolic/vascular	0,34933333
MSRS_deep_occipital	HDL cholesterol	0,144	0,337	Metabolic/vascular	0,44933333
MSRS_deep_occipital	LDL cholesterol	0,225	0,131	Metabolic/vascular	0,34933333
MSRS_deep_occipital	TGC	0,194	0,194	Metabolic/vascular	0,388
MSRS_deep_occipital	Total cholesterol	0,241	0,106	Metabolic/vascular	0,34933333
MSRS_deep_occipital	Vitamin B12	0,034	0,817	Metabolic/vascular	0,817
MSRS_deep_occipital	Vitamin D	0,155	0,325	Metabolic/vascular	0,44933333
MSRS_deep_parietal	Folic acid levels	0,16	0,286	Metabolic/vascular	0,854
MSRS_deep_parietal	Glycate hemoglobin	0,125	0,427	Metabolic/vascular	0,854
MSRS_deep_parietal	HDL cholesterol	0,0772	0,609	Metabolic/vascular	0,9744
MSRS_deep_parietal	LDL cholesterol	-0,005	0,971	Metabolic/vascular	0,987

MSRS_deep_parietal	TGC	0,023	0,877	Metabolic/vascular	0,987
MSRS_deep_parietal	Total cholesterol	-0,002	0,987	Metabolic/vascular	0,987
MSRS_deep_parietal	Vitamin B12	0,122	0,416	Metabolic/vascular	0,854
MSRS_deep_parietal	Vitamin D	0,215	0,169	Metabolic/vascular	0,854
MSRS_deep_temporal	Folic acid levels	0,198	0,184	Metabolic/vascular	0,72266667
MSRS_deep_temporal	Glycate hemoglobin	0,286	0,066	Metabolic/vascular	0,528
MSRS_deep_temporal	HDL cholesterol	-0,096	0,523	Metabolic/vascular	0,8368
MSRS_deep_temporal	LDL cholesterol	-0,039	0,793	Metabolic/vascular	0,884
MSRS_deep_temporal	TGC	0,118	0,432	Metabolic/vascular	0,8368
MSRS_deep_temporal	Total cholesterol	-0,022	0,884	Metabolic/vascular	0,884
MSRS_deep_temporal	Vitamin B12	0,165	0,271	Metabolic/vascular	0,72266667
MSRS_deep_temporal	Vitamin D	-0,067	0,669	Metabolic/vascular	0,884
MSRS_deep_total	Folic acid levels	0,139	0,354	Metabolic/vascular	0,91066667
MSRS_deep_total	Glycate hemoglobin	0,254	0,103	Metabolic/vascular	0,824
MSRS_deep_total	HDL cholesterol	0,082	0,584	Metabolic/vascular	0,91066667
MSRS_deep_total	LDL cholesterol	0,001	0,993	Metabolic/vascular	0,993
MSRS_deep_total	TGC	0,09	0,55	Metabolic/vascular	0,91066667
MSRS_deep_total	Total cholesterol	0,024	0,871	Metabolic/vascular	0,993
MSRS_deep_total	Vitamin B12	0,061	0,683	Metabolic/vascular	0,91066667
MSRS_deep_total	Vitamin D	0,091	0,565	Metabolic/vascular	0,91066667
MSRS_total	Folic acid levels	0,245	0,1	Metabolic/vascular	0,4
MSRS_total	Glycate hemoglobin	0,274	0,078	Metabolic/vascular	0,4

MSRS_total	HDL cholesterol	0,0317	0,834	Metabolic/vascular	0,916
MSRS_total	LDL cholesterol	-0,033	0,824	Metabolic/vascular	0,916
MSRS_total	TGC	0,106	0,479	Metabolic/vascular	0,916
MSRS_total	Total cholesterol	-0,015	0,916	Metabolic/vascular	0,916
MSRS_total	Vitamin B12	0,121	0,422	Metabolic/vascular	0,916
MSRS_total	Vitamin D	0,065	0,679	Metabolic/vascular	0,916

Benjamini–Hochberg FDR (False Discovery Rate) correction applied within families of tests defined by categories – Metabolic/vascular category. The significance level adopted was p<0.05 after correction (Sig.).

**WMH_metric**	**Variable**	**tau**	**Sig.**	**Category**	**Sig.**
Fazekas	FFT	-0,319	0,03	Motor/Psychomotor	**0,03**
Fazekas	SPES	0,433	0,003	Motor/Psychomotor	**0,006**
MSRS_PV_frontal	FFT	-0,089	0,552	Motor/Psychomotor	0,552
MSRS_PV_frontal	SPES	0,241	0,114	Motor/Psychomotor	0,228
MSRS_PV_lateral	FFT	-0,073	0,625	Motor/Psychomotor	0,625
MSRS_PV_lateral	SPES	0,308	0,041	Motor/Psychomotor	0,082
MSRS_PV_occipital	FFT	-0,025	0,864	Motor/Psychomotor	0,864
MSRS_PV_occipital	SPES	0,377	0,011	Motor/Psychomotor	**0,022**
MSRS_PV_total	FFT	-0,046	0,756	Motor/Psychomotor	0,756
MSRS_PV_total	SPES	0,391	0,008	Motor/Psychomotor	**0,016**
MSRS_deep_frontal	FFT	-0,196	0,191	Motor/Psychomotor	0,191
MSRS_deep_frontal	SPES	0,421	0,004	Motor/Psychomotor	**0,008**

MSRS_deep_occipital	FFT	-0,107	0,478	Motor/Psychomotor	0,85
MSRS_deep_occipital	SPES	0,029	0,85	Motor/Psychomotor	0,85
MSRS_deep_parietal	FFT	-0,112	0,456	Motor/Psychomotor	0,456
MSRS_deep_parietal	SPES	0,428	0,003	Motor/Psychomotor	**0,006**
MSRS_deep_temporal	FFT	-0,032	0,827	Motor/Psychomotor	0,827
MSRS_deep_temporal	SPES	0,448	0,002	Motor/Psychomotor	**0,004**
MSRS_deep_total	FFT	-0,159	0,289	Motor/Psychomotor	0,289
MSRS_deep_total	SPES	0,419	0,004	Motor/Psychomotor	**0,008**
MSRS_total	FFT	-0,124	0,411	Motor/Psychomotor	0,411
MSRS_total	SPES	0,402	0,006	Motor/Psychomotor	**0,012**

Benjamini–Hochberg FDR (False Discovery Rate) correction applied within families of tests defined by categories – Motor/psychomotor category. The significance level adopted was p<0.05 after correction (Sig.).

**WMH_metric**	**Variable**	**tau**	**Sig.**	**Category**	**Sig.**
Fazekas	Agitation	-0,095	0,526	Neuropsychiatric	0,7574
Fazekas	Anxiety	-0,01	0,943	Neuropsychiatric	0,943
Fazekas	Apathy	0,16	0,285	Neuropsychiatric	0,665
Fazekas	Appetite	-0,092	0,541	Neuropsychiatric	0,7574
Fazekas	Delusions	-0,441	0,002	Neuropsychiatric	**0,028**
Fazekas	Depression/dysphoria	0,077	0,608	Neuropsychiatric	0,76138462
Fazekas	Disinhibition	-0,141	0,349	Neuropsychiatric	0,698
Fazekas	Euphoria	-0,056	0,707	Neuropsychiatric	0,76138462

Fazekas	Hallucinations	-0,209	0,161	Neuropsychiatric	0,4508
Fazekas	Irritability	-0,217	0,146	Neuropsychiatric	0,4508
Fazekas	Motor behavior	0,066	0,661	Neuropsychiatric	0,76138462
Fazekas	NPI total	-0,242	0,104	Neuropsychiatric	0,4508
Fazekas	Nocturnal behavior	-0,112	0,456	Neuropsychiatric	0,7574
Fazekas	SBQ-R	0,238	0,11	Neuropsychiatric	0,4508
MSRS_PV_frontal	Agitation	0,106	0,482	Neuropsychiatric	0,8344
MSRS_PV_frontal	Anxiety	0,026	0,858	Neuropsychiatric	0,93476923
MSRS_PV_frontal	Apathy	-0,191	0,201	Neuropsychiatric	0,8344
MSRS_PV_frontal	Appetite	-0,088	0,556	Neuropsychiatric	0,8344
MSRS_PV_frontal	Delusions	-0,131	0,384	Neuropsychiatric	0,8344
MSRS_PV_frontal	Depression/dysphoria	-0,085	0,571	Neuropsychiatric	0,8344
MSRS_PV_frontal	Disinhibition	-0,025	0,868	Neuropsychiatric	0,93476923
MSRS_PV_frontal	Euphoria	0,08	0,596	Neuropsychiatric	0,8344
MSRS_PV_frontal	Hallucinations	-0,003	0,981	Neuropsychiatric	0,981
MSRS_PV_frontal	Irritability	-0,111	0,46	Neuropsychiatric	0,8344
MSRS_PV_frontal	Motor behavior	0,226	0,13	Neuropsychiatric	0,8344
MSRS_PV_frontal	NPI total	-0,088	0,559	Neuropsychiatric	0,8344
MSRS_PV_frontal	Nocturnal behavior	-0,061	0,683	Neuropsychiatric	0,86927273
MSRS_PV_frontal	SBQ-R	0,231	0,121	Neuropsychiatric	0,8344
MSRS_PV_lateral	Agitation	0,083	0,58	Neuropsychiatric	0,812
MSRS_PV_lateral	Anxiety	-0,004	0,975	Neuropsychiatric	0,975

MSRS_PV_lateral	Apathy	-0,117	0,436	Neuropsychiatric	0,763
MSRS_PV_lateral	Appetite	-0,124	0,408	Neuropsychiatric	0,763
MSRS_PV_lateral	Delusions	-0,211	0,158	Neuropsychiatric	0,553
MSRS_PV_lateral	Depression/dysphoria	-0,126	0,4	Neuropsychiatric	0,763
MSRS_PV_lateral	Disinhibition	0,17	0,258	Neuropsychiatric	0,7224
MSRS_PV_lateral	Euphoria	0,213	0,155	Neuropsychiatric	0,553
MSRS_PV_lateral	Hallucinations	-0,265	0,074	Neuropsychiatric	0,553
MSRS_PV_lateral	Irritability	0,038	0,8	Neuropsychiatric	0,96492308
MSRS_PV_lateral	Motor behavior	0,261	0,079	Neuropsychiatric	0,553
MSRS_PV_lateral	NPI total	-0,085	0,574	Neuropsychiatric	0,812
MSRS_PV_lateral	Nocturnal behavior	-0,019	0,896	Neuropsychiatric	0,96492308
MSRS_PV_lateral	SBQ-R	0,027	0,855	Neuropsychiatric	0,96492308
MSRS_PV_occipital	Agitation	-0,14	0,35	Neuropsychiatric	0,901
MSRS_PV_occipital	Anxiety	-0,021	0,889	Neuropsychiatric	0,901
MSRS_PV_occipital	Apathy	-0,05	0,737	Neuropsychiatric	0,901
MSRS_PV_occipital	Appetite	-0,265	0,074	Neuropsychiatric	0,518
MSRS_PV_occipital	Delusions	-0,386	0,008	Neuropsychiatric	0,112
MSRS_PV_occipital	Depression/dysphoria	-0,018	0,901	Neuropsychiatric	0,901
MSRS_PV_occipital	Disinhibition	0,036	0,81	Neuropsychiatric	0,901
MSRS_PV_occipital	Euphoria	0,06	0,688	Neuropsychiatric	0,901
MSRS_PV_occipital	Hallucinations	-0,13	0,388	Neuropsychiatric	0,901
MSRS_PV_occipital	Irritability	-0,088	0,56	Neuropsychiatric	0,901
MSRS_PV_occipital	Motor behavior	-0,04	0,788	Neuropsychiatric	0,901

MSRS_PV_occipital	NPI total	-0,238	0,111	Neuropsychiatric	0,518
MSRS_PV_occipital	Nocturnal behavior	-0,078	0,605	Neuropsychiatric	0,901
MSRS_PV_occipital	SBQ-R	0,156	0,298	Neuropsychiatric	0,901
MSRS_PV_total	Agitation	0,003	0,982	Neuropsychiatric	0,982
MSRS_PV_total	Anxiety	-0,038	0,798	Neuropsychiatric	0,85938462
MSRS_PV_total	Apathy	-0,128	0,393	Neuropsychiatric	0,68775
MSRS_PV_total	Appetite	-0,181	0,227	Neuropsychiatric	0,6356
MSRS_PV_total	Delusions	-0,318	0,031	Neuropsychiatric	0,434
MSRS_PV_total	Depression/dysphoria	-0,105	0,486	Neuropsychiatric	0,73966667
MSRS_PV_total	Disinhibition	0,078	0,601	Neuropsychiatric	0,73966667
MSRS_PV_total	Euphoria	0,142	0,345	Neuropsychiatric	0,68775
MSRS_PV_total	Hallucinations	-0,203	0,176	Neuropsychiatric	0,6356
MSRS_PV_total	Irritability	-0,071	0,634	Neuropsychiatric	0,73966667
MSRS_PV_total	Motor behavior	0,187	0,212	Neuropsychiatric	0,6356
MSRS_PV_total	NPI total	-0,196	0,191	Neuropsychiatric	0,6356
MSRS_PV_total	Nocturnal behavior	-0,076	0,614	Neuropsychiatric	0,73966667
MSRS_PV_total	SBQ-R	0,141	0,349	Neuropsychiatric	0,68775
MSRS_deep_frontal	Agitation	-0,154	0,304	Neuropsychiatric	0,608
MSRS_deep_frontal	Anxiety	-0,174	0,246	Neuropsychiatric	0,574
MSRS_deep_frontal	Apathy	0,136	0,363	Neuropsychiatric	0,63525
MSRS_deep_frontal	Appetite	0,036	0,808	Neuropsychiatric	0,932
MSRS_deep_frontal	Delusions	-0,352	0,016	Neuropsychiatric	0,189

MSRS_deep_frontal	Depression/dysphoria	0,012	0,932	Neuropsychiatric	0,932
MSRS_deep_frontal	Disinhibition	-0,119	0,428	Neuropsychiatric	0,66577778
MSRS_deep_frontal	Euphoria	-0,071	0,637	Neuropsychiatric	0,81072727
MSRS_deep_frontal	Hallucinations	-0,084	0,575	Neuropsychiatric	0,805
MSRS_deep_frontal	Irritability	-0,324	0,027	Neuropsychiatric	0,189
MSRS_deep_frontal	Motor behavior	0,221	0,139	Neuropsychiatric	0,3892
MSRS_deep_frontal	NPI total	-0,243	0,102	Neuropsychiatric	0,357
MSRS_deep_frontal	Nocturnal behavior	-0,024	0,872	Neuropsychiatric	0,932
MSRS_deep_frontal	SBQ-R	0,25	0,093	Neuropsychiatric	0,357
MSRS_deep_occipital	Agitation	-0,093	0,536	Neuropsychiatric	0,73054545
MSRS_deep_occipital	Anxiety	0,179	0,231	Neuropsychiatric	0,71225
MSRS_deep_occipital	Apathy	-0,124	0,407	Neuropsychiatric	0,71225
MSRS_deep_occipital	Appetite	-0,177	0,237	Neuropsychiatric	0,71225
MSRS_deep_occipital	Delusions	0,012	0,935	Neuropsychiatric	0,992
MSRS_deep_occipital	Depression/dysphoria	0,02	0,891	Neuropsychiatric	0,992
MSRS_deep_occipital	Disinhibition	-0,158	0,292	Neuropsychiatric	0,71225
MSRS_deep_occipital	Euphoria	-0,151	0,313	Neuropsychiatric	0,71225
MSRS_deep_occipital	Hallucinations	0,126	0,4	Neuropsychiatric	0,71225
MSRS_deep_occipital	Irritability	-0,171	0,254	Neuropsychiatric	0,71225
MSRS_deep_occipital	Motor behavior	0,001	0,992	Neuropsychiatric	0,992
MSRS_deep_occipital	NPI total	-0,111	0,46	Neuropsychiatric	0,71555556
MSRS_deep_occipital	Nocturnal behavior	0,084	0,574	Neuropsychiatric	0,73054545
MSRS_deep_occipital	SBQ-R	0,251	0,092	Neuropsychiatric	0,71225

MSRS_deep_parietal	Agitation	-0,336	0,022	Neuropsychiatric	0,098
MSRS_deep_parietal	Anxiety	-0,154	0,304	Neuropsychiatric	0,497
MSRS_deep_parietal	Apathy	0,076	0,614	Neuropsychiatric	0,71633333
MSRS_deep_parietal	Appetite	-0,139	0,355	Neuropsychiatric	0,497
MSRS_deep_parietal	Delusions	-0,31	0,035	Neuropsychiatric	0,098
MSRS_deep_parietal	Depression/dysphoria	-0,007	0,962	Neuropsychiatric	0,964
MSRS_deep_parietal	Disinhibition	-0,204	0,171	Neuropsychiatric	0,399
MSRS_deep_parietal	Euphoria	-0,142	0,345	Neuropsychiatric	0,497
MSRS_deep_parietal	Hallucinations	-0,104	0,488	Neuropsychiatric	0,62109091
MSRS_deep_parietal	Irritability	-0,331	0,024	Neuropsychiatric	0,098
MSRS_deep_parietal	Motor behavior	-0,006	0,964	Neuropsychiatric	0,964
MSRS_deep_parietal	NPI total	-0,431	0,002	Neuropsychiatric	0,028
MSRS_deep_parietal	Nocturnal behavior	-0,19	0,205	Neuropsychiatric	0,41
MSRS_deep_parietal	SBQ-R	0,313	0,033	Neuropsychiatric	0,098
MSRS_deep_temporal	Agitation	-0,122	0,416	Neuropsychiatric	0,884
MSRS_deep_temporal	Anxiety	0,034	0,82	Neuropsychiatric	0,884
MSRS_deep_temporal	Apathy	-0,226	0,129	Neuropsychiatric	0,602
MSRS_deep_temporal	Appetite	-0,064	0,668	Neuropsychiatric	0,884
MSRS_deep_temporal	Delusions	-0,08	0,596	Neuropsychiatric	0,884
MSRS_deep_temporal	Depression/dysphoria	0,057	0,704	Neuropsychiatric	0,884
MSRS_deep_temporal	Disinhibition	-0,028	0,851	Neuropsychiatric	0,884
MSRS_deep_temporal	Euphoria	0,022	0,884	Neuropsychiatric	0,884

MSRS_deep_temporal	Hallucinations	-0,13	0,385	Neuropsychiatric	0,884
MSRS_deep_temporal	Irritability	-0,228	0,126	Neuropsychiatric	0,602
MSRS_deep_temporal	Motor behavior	0,03	0,841	Neuropsychiatric	0,884
MSRS_deep_temporal	NPI total	-0,14	0,352	Neuropsychiatric	0,884
MSRS_deep_temporal	Nocturnal behavior	-0,093	0,534	Neuropsychiatric	0,884
MSRS_deep_temporal	SBQ-R	0,341	0,02	Neuropsychiatric	0,28
MSRS_deep_total	Agitation	-0,243	0,102	Neuropsychiatric	0,2856
MSRS_deep_total	Anxiety	-0,131	0,384	Neuropsychiatric	0,60709091
MSRS_deep_total	Apathy	0,107	0,477	Neuropsychiatric	0,60709091
MSRS_deep_total	Appetite	-0,007	0,957	Neuropsychiatric	0,957
MSRS_deep_total	Delusions	-0,348	0,017	Neuropsychiatric	0,10733333
MSRS_deep_total	Depression/dysphoria	0,019	0,896	Neuropsychiatric	0,957
MSRS_deep_total	Disinhibition	-0,163	0,278	Neuropsychiatric	0,60709091
MSRS_deep_total	Euphoria	-0,114	0,449	Neuropsychiatric	0,60709091
MSRS_deep_total	Hallucinations	-0,11	0,463	Neuropsychiatric	0,60709091
MSRS_deep_total	Irritability	-0,362	0,013	Neuropsychiatric	0,10733333
MSRS_deep_total	Motor behavior	0,142	0,344	Neuropsychiatric	0,60709091
MSRS_deep_total	NPI total	-0,315	0,032	Neuropsychiatric	0,112
MSRS_deep_total	Nocturnal behavior	-0,084	0,577	Neuropsychiatric	0,67316667
MSRS_deep_total	SBQ-R	0,333	0,023	Neuropsychiatric	0,10733333
MSRS_total	Agitation	-0,166	0,267	Neuropsychiatric	0,623
MSRS_total	Anxiety	-0,071	0,637	Neuropsychiatric	0,81072727
MSRS_total	Apathy	0,05	0,738	Neuropsychiatric	0,861

MSRS_total	Appetite	-0,072	0,631	Neuropsychiatric	0,81072727
MSRS_total	Delusions	-0,383	0,008	Neuropsychiatric	0,112
MSRS_total	Depression/dysphoria	-0,008	0,956	Neuropsychiatric	0,956
MSRS_total	Disinhibition	-0,077	0,608	Neuropsychiatric	0,81072727
MSRS_total	Euphoria	-0,024	0,871	Neuropsychiatric	0,938
MSRS_total	Hallucinations	-0,19	0,204	Neuropsychiatric	0,5712
MSRS_total	Irritability	-0,286	0,053	Neuropsychiatric	0,224
MSRS_total	Motor behavior	0,149	0,32	Neuropsychiatric	0,64
MSRS_total	NPI total	-0,286	0,053	Neuropsychiatric	0,224
MSRS_total	Nocturnal behavior	-0,085	0,571	Neuropsychiatric	0,81072727
MSRS_total	SBQ-R	0,274	0,064	Neuropsychiatric	0,224

Benjamini–Hochberg FDR (False Discovery Rate) correction applied within families of tests defined by categories – Neuropsychiatric category. The significance level adopted was p<0.05 after correction (Sig.).

Supplementary material S9.

**WMH measure**	**Clinical domain**	**Variable**	**Schizophrenia spectrum disorders, N=11 (τ, p)**	**Bipolar disorder, N=17 (τ, p)**	**Major Depressive disorder, N=18 (τ, p)**	**Directional consistency**
Fazekas scale	Cognitive	MoCA	-0.24, 0.353	-0.36, 0.074	-0.39, 0.055	Yes
Fazekas scale	Cognitive	MoCA visuospatial/executive	-0.07, 0.779	-0.24, 0.257	-0.31, 0.127	Yes
Fazekas scale	Cognitive	MoCA abstraction	0.14, 0.612	-0.53, 0.018	-0.21, 0.329	No
MSRSpv lateral walls	Cognitive	MoCA abstraction	0.17, 0.557	-0.47, 0.034	-0.31, 0.158	No

Fazekas scale	Cognitive	MoCA memory	-0.05, 0.850	-0.47, 0.029	-0.31, 0.144	Yes
Fazekas scale	Cognitive	MoCA orientation	-0.55, 0.066	-0.09, 0.683	-0.44, 0.049	Yes
MSRS	Cognitive	MoCA orientation	-0.45, 0.107	-0.12, 0.548	-0.45, 0.028	Yes
MSRSd	Cognitive	MoCA orientation	-0.47, 0.096	-0.08, 0.700	-0.42, 0.042	Yes
MSRSd frontal	Cognitive	MoCA orientation	-0.50, 0.084	-0.08, 0.696	-0.38, 0.069	Yes
MSRSd parietal	Cognitive	MoCA orientation	-0.50, 0.084	-0.18, 0.433	-0.36, 0.087	Yes
MSRSd temporal	Cognitive	MoCA orientation	.	-0.27, 0.261	-0.63, 0.006	Yes
MSRSpv frontal	Cognitive	MoCA orientation	-0.42, 0.162	-0.20, 0.380	-0.47, 0.042	Yes
MSRSpv lateral walls	Cognitive	MoCA orientation	-0.54, 0.076	-0.21, 0.354	-0.42, 0.071	Yes
MSRSpv occipital	Cognitive	MoCA orientation	-0.56, 0.064	-0.19, 0.426	-0.24, 0.289	Yes
Fazekas scale	Cognitive	MMSE orientation	-0.22, 0.399	-0.07, 0.733	-0.44, 0.049	Yes
MSRSd temporal	Cognitive	MMSE orientation	.	-0.05, 0.814	-0.63, 0.006	Yes
Fazekas scale	Psychomotor	FTT	-0.26, 0.313	-0.21, 0.292	-0.03, 0.860	Yes
Fazekas scale	Psychomotor	SPES	0.20, 0.441	0.25, 0.223	0.705, 0.001	Yes
MSRS	Psychomotor	SPES	-0.06, 0.801	0.28, 0.145	0.639, 0.001	No
MSRSd	Psychomotor	SPES	0.18, 0.487	0.30, 0.123	0.58, 0.003	Yes
MSRSpv	Psychomotor	SPES	-0.15, 0.557	0.27, 0.163	0.66, 0.001	No
MSRSd frontal	Psychomotor	SPES	0.24, 0.365	0.19, 0.348	0.67, 0.001	Yes
MSRSd parietal	Psychomotor	SPES	0.12, 0.651	0.43, 0.036	0.47, 0.021	Yes
MSRSd temporal	Psychomotor	SPES	.	0.39, 0.071	0.50, 0.023	Yes
MSRSpv lateral walls	Psychomotor	SPES	-0.12, 0.642	0.12, 0.56	0.654, 0.003	Yes
MSRSd temporal	Psychopathology	Psychiatric hospitalizations	.	0.39, 0.071	0.30, 0.172	Yes

MSRSd	Psychopathology	NPI	-0.11, 0.633	0.04, 0.826	-0.43, 0.022	No
MSRSd parietal	Psychopathology	NPI	-0.52, 0.039	-0.22, 0.262	-0.33, 0.075	Yes
Fazekas scale	Psychopathology	NPI delusions	-0.78, 0.007	-0.14, 0.504	-0.06, 0.763	Yes
MSRS	Psychopathology	NPI delusions	-0.56, 0.040	-0.17, 0.382	-0.18, 0.368	Yes
MSRSd	Psychopathology	NPI delusions	-0.35, 0.204	-0.11, 0.559	-0.18, 0.365	Yes
MSRSpv	Psychopathology	NPI delusions	-0.61, 0.024	-0.24, 0.234	-0.03, 0.870	Yes
MSRSd frontal	Psychopathology	NPI delusions	-0.40, 0.152	-0.22, 0.275	-0.13, 0.517	Yes
MSRSd parietal	Psychopathology	NPI delusions	-0.40, 0.152	-0.03, 0.879	-0.24, 0.252	Yes
MSRSpv occipital	Psychopathology	NPI delusions	-0.81, 0.007	-0.09, 0.679	-0.42, 0.059	Yes
MSRSd	Psychopathology	NPI irritability	-0.28, 0.289	-0.27, 0.178	-0.23, 0.256	Yes
MSRSd frontal	Psychopathology	NPI irritability	-0.22, 0.423	-0.24, 0.237	-0.29, 0.164	Yes
MSRSd parietal	Psychopathology	NPI irritability	-0.22, 0.423	-0.40, 0.060	-0.10, 0.625	Yes
MSRSd	Psychopathology	SBQ-R	0.54, 0.044	0.11, 0.575	-0.01, 0.938	Partial
MSRSd parietal	Psychopathology	SBQ-R	0.63, 0.022	0.20, 0.342	0.007, 0.968	Yes
MSRSd temporal	Psychopathology	SBQ-R	.	0.49, 0.029	0.13, 0.511	Yes
MSRS	Neuroimaging	GCA-F	0.13, 0.606	0.10, 0.622	0.370, 0.067	Yes
MSRSpv	Metabolic	HbA1c	0.65, 0.024	-0.03, 0.860	0.22, 0.221	Partial

Sensitivity analysis. Direction and consistency of Kendall's tau correlation analysis between multiple variables of the subgroup of patients with disease onset at age 40 or more, stratified by diagnosis (Schizophrenia spectrum disorders, Bipolar disorder, Major Depressive disorder). τ- Kendall's tau correlation coefficient, p – significance level, MSRS - Modified Scheltens rating scale, MSRSpv - Modified Scheltens rating scale periventricular lesions, MSRSd - Modified Scheltens rating scale deep lesions, MoCA – Montreal Cognitive assessment total score, MMSE – Mini mental state examination total score, FTT – Finger tapping test, FAB – Frontal assessment battery, SPES – Short Parkinson evaluation scale, NPI – Neuropsychiatric inventory total score, SBQ-R – Suicide Behaviors Questionnaire-Revised, MTA – Medial temporal atrophy scale, GCA-F – Global cortical atrophy/Pasquier scale frontal subscale.

Supplementary material S10.

WMH measure	Clinical domain	Variable	Onset ≥50y N=27, (τ, p)	Directional consistency ≥50 vs. ≥40y	Onset ≥60y N=11, (τ, p)	Directional consistency ≥60 vs. ≥40y
Fazekas scale	Cognitive	MoCA	-0.39, 0.013	Yes	-0.25, 0.334	Yes
Fazekas scale	Cognitive	MoCA visuospatial/executive	-0.20, 0.215	Yes	0.30, 0.269	No
Fazekas scale	Cognitive	MoCA abstraction	-0.23, 0.189	Yes	0.105, 0.709	No
MSRSpv lateral walls	Cognitive	MoCA abstraction	-0.22, 0.212	Yes	-0.05, 0.843	Yes
Fazekas scale	Cognitive	MoCA memory	-0.26, 0.118	Yes	-0.05, 0.854	Yes
Fazekas scale	Cognitive	MoCA orientation	-0.40, 0.026	Yes	-0.50, 0.503	Yes
MSRS	Cognitive	MoCA orientation	-0.39, 0.016	Yes	-0.47, 0.70	Yes
MSRSd	Cognitive	MoCA orientation	-0.39, 0.018	Yes	-0.480, 0.069	Yes
MSRSd frontal	Cognitive	MoCA orientation	-0.37, 0.028	Yes	-0.41, 0.125	Yes
MSRSd parietal	Cognitive	MoCA orientation	-0.40, 0.018	Yes	-0.38, 0.152	Yes
MSRSd temporal	Cognitive	MoCA orientation	-0.24, 0.203	Yes	-0.48, 0.108	Yes
MSRSpv frontal	Cognitive	MoCA orientation	-0.32, 0.086	Yes	-0.32, 0.267	Yes
MSRSpv lateral walls	Cognitive	MoCA orientation	-0.42, 0.022	Yes	-0.55, 0.59	Yes
MSRSpv occipital	Cognitive	MoCA orientation	-0.18, 0.323	Yes	-0.15, 0.588	Yes
Fazekas scale	Cognitive	MMSE orientation	-0.33, 0.062	Yes	-0.34, 0.225	Yes
MSRSd temporal	Cognitive	MMSE orientation	-0.17, 0.362	Yes	-0.55, 0.066	Yes
Fazekas scale	Psychomotor	FTT	-0.35, 0.025	Yes	-0.15, 0.542	Yes
Fazekas scale	Psychomotor	SPES	0.38, 0.020	Yes	0.24, 0.376	Yes
MSRS	Psychomotor	SPES	0.34, 0.021	Yes	0.04, 0.856	Yes
MSRSd	Psychomotor	SPES	0.33, 0.027	Yes	0.11, 0.650	Yes
MSRSpv	Psychomotor	SPES	0.34, 0.028	Yes	0.04, 0.854	Yes

MSRSd frontal	Psychomotor	SPES	0.33, 0.032	Yes	0.14, 0.580	Yes
MSRSd parietal	Psychomotor	SPES	0.34, 0.029	Yes	0.22, 0.401	Yes
MSRSd temporal	Psychomotor	SPES	0.38, 0.028	Yes	0.45, 0.115	Yes
MSRSpv lateral walls	Psychomotor	SPES	0.34, 0.041	Yes	0.26, 0.348	Yes
MSRSd temporal	Psychopathology	Psychiatric hospitalizations	0.31, 0.098	Yes	.	.
MSRSd	Psychopathology	NPI	-0.35, 0.017	Yes	-0.54, 0.025	Yes
MSRSd parietal	Psychopathology	NPI	-0.54, <0.001	Yes	-0.66, 0.007	Yes
Fazekas scale	Psychopathology	NPI delusions	-0.51, 0.003	Yes	-0.35, 0.197	Yes
MSRS	Psychopathology	NPI delusions	-0.41, 0.009	Yes	-0.57, 0.024	Yes
MSRSd	Psychopathology	NPI delusions	-0.36, 0.024	Yes	-0.51, 0.044	Yes
MSRSpv	Psychopathology	NPI delusions	-0.37, 0.024	Yes	-0.46, 0.074	Yes
MSRSd frontal	Psychopathology	NPI delusions	-0.32, 0.048	Yes	-0.37, 0.148	Yes
MSRSd parietal	Psychopathology	NPI delusions	-0.42, 0.011	Yes	-0.53, 0.041	Yes
MSRSpv occipital	Psychopathology	NPI delusions	-0.01, 0.945	Yes	-0.65, 0.019	Yes
MSRSd	Psychopathology	NPI irritability	-0.39, 0.012	Yes	-0.37, 0.141	Yes
MSRSd frontal	Psychopathology	NPI irritability	-0.32, 0.044	Yes	-0.18, 0.482	Yes
MSRSd parietal	Psychopathology	NPI irritability	-0.41, 0.012	Yes	-0.32, 0.219	Yes
MSRSd	Psychopathology	SBQ-R	0.21, 0.154	Yes	0.29, 0.245	Yes
MSRSd parietal	Psychopathology	SBQ-R	0.29, 0.061	Yes	0.36, 0.151	Yes
MSRSd temporal	Psychopathology	SBQ-R	0.34, 0.05	Yes	0.47, 0.96	Yes
MSRS	Neuroimaging	GCA-F	0.22, 0.169	Yes	0.10, 0.704	Yes
MSRSpv	Metabolic	HbA1c	0.27, 0.086	Yes	0.31, 0.227	Yes

Sensitivity analysis. Kendall's tau correlation analysis in patients with disease onset at age 50 or more and age 60 or more. τ- Kendall's tau correlation coefficient, p – significance level, MSRS - Modified Scheltens rating scale, MSRSpv - Modified Scheltens rating scale periventricular lesions, MSRSd - Modified Scheltens rating scale deep lesions, MoCA – Montreal Cognitive assessment total score, MMSE – Mini mental state examination total score, FTT – Finger tapping test, FAB – Frontal assessment battery, SPES – Short Parkinson evaluation scale, NPI – Neuropsychiatric inventory total score, SBQ-R – Suicide Behaviors Questionnaire-Revised, MTA – Medial temporal atrophy scale, GCA-F – Global cortical atrophy/Pasquier scale frontal subscale.

Supplementary material S11.

						95% Confidence Interval	
Predictors	B	Std. Error	Standardized Coefficients Beta	t	Sig.	Lower Bound	Upper Bound
(Constant)	1.382	.662		2.089	.043	.047	2.718
Age	-.020	.011	-.245	-1.744	.088	-.043	.003
Vascular Composite	.026	.048	.068	.541	.592	-.071	.122
FAZEKAS grade	-.364	.107	-.495	-3.410	.001	-.580	-.149

Multiple linear regression model conducted in the subgroup of patients with a disease onset ≥ 40 years to examine whether age, vascular burden and WMH burden was associated with the cognitive-psychopathologic composite index (MoCA visuospatial/executive, MoCA orientation, MoCA memory, NPI delusions, and SBQ-R).

Supplementary material S12.

Variables	Onset <40y N=44	Onset ≥40y N=46	Stat.(1)	Sig.(2)
Treatment with typical antipsychotics, %	15.9	4.3	3.33	0.087
Treatment with atypical antipsychotics, %	93.2	87	0.96	0.486
Treatment with lithium, %	20.5	4.3	5.43	0.025
Treatment with valproate/valproic acid, %	31.8	19.6	1.77	0.230
Treatment with benzodiazepines, %	88.6	91.3	0.178	0.737
Treatment with tricyclic antidepressants, %	6.8	4.3	0.262	0.673
Treatment with SSRI, %	36.4	50	1.70	0.209
Treatment with psychostimulants, %	6.8	2.2	1.42	0.35

Comparison of the proportion of psychopharmacological treatments between the patients with an onset before 40 years and ate 40 or more. (1) chi-square statistics, (2) significance level.
